# Safety, immunogenicity, and protection provided by unadjuvanted and adjuvanted formulations of a recombinant plant-derived virus-like particle vaccine candidate for COVID-19 in nonhuman primates

**DOI:** 10.1038/s41423-021-00809-2

**Published:** 2022-01-05

**Authors:** Stéphane Pillet, Prabhu S. Arunachalam, Guadalupe Andreani, Nadia Golden, Jane Fontenot, Pyone Pyone Aye, Katharina Röltgen, Gabrielle Lehmicke, Philipe Gobeil, Charlotte Dubé, Sonia Trépanier, Nathalie Charland, Marc-André D’Aoust, Kasi Russell-Lodrigue, Christopher Monjure, Robert V. Blair, Scott D. Boyd, Rudolf P. Bohm, Jay Rappaport, François Villinger, Nathalie Landry, Bali Pulendran, Brian J. Ward

**Affiliations:** 1 Medicago Inc., Québec, QC Canada; 2 Institute for Immunity, Transplantation and Infection, Stanford University School of Medicine, Stanford University, Stanford, CA USA; 3 Tulane National Primate Research Center, Covington, LA USA; 4 New Iberia Research Center, University of Louisiana at Lafayette, New Iberia, LA USA; 5 Department of Pathology, Stanford University School of Medicine, Stanford University, Stanford, CA USA; 6 Department of Microbiology and Immunology, Tulane University School of Medicine, New Orleans, LA USA; 7 Department of Microbiology and Immunology, Stanford University School of Medicine, Stanford University, Stanford, CA USA; 8 Institute for Immunity, Transplantation & Infection, Stanford University School of Medicine, Stanford University, Stanford, CA USA; 9 Research Institute of the McGill University Health Centre, Montreal, QC Canada

**Keywords:** SARS-CoV-2, Virus-like particles, Non-humane primates, AS03, CpG1018, Protein vaccines, Adjuvants

## Abstract

Although antivirals are important tools to control severe acute respiratory syndrome coronavirus 2 (SARS-CoV-2) infection, effective vaccines are essential to control the current coronavirus disease 2019 (COVID-19) pandemic. Plant-derived virus-like particle (VLP) vaccine candidates have previously demonstrated immunogenicity and efficacy against influenza. Here, we report the immunogenicity and protection induced in rhesus macaques by intramuscular injections of a VLP bearing a SARS-CoV-2 spike protein (CoVLP) vaccine candidate formulated with or without Adjuvant System 03 (AS03) or cytidine-phospho-guanosine (CpG) 1018. Although a single dose of the unadjuvanted CoVLP vaccine candidate stimulated humoral and cell-mediated immune responses, booster immunization (at 28 days after priming) and adjuvant administration significantly improved both responses, with higher immunogenicity and protection provided by the AS03-adjuvanted CoVLP. Fifteen micrograms of CoVLP adjuvanted with AS03 induced a polyfunctional interleukin-2 (IL-2)-driven response and IL-4 expression in CD4 T cells. Animals were challenged by multiple routes (i.e., intratracheal, intranasal, and ocular) with a total viral dose of 10^6^ plaque-forming units of SARS-CoV-2. Lower viral replication in nasal swabs and bronchoalveolar lavage fluid (BALF) as well as fewer SARS-CoV-2-infected cells and immune cell infiltrates in the lungs concomitant with reduced levels of proinflammatory cytokines and chemotactic factors in the BALF were observed in animals immunized with the CoVLP adjuvanted with AS03. No clinical, pathologic, or virologic evidence of vaccine-associated enhanced disease was observed in vaccinated animals. The CoVLP adjuvanted with AS03 was therefore selected for vaccine development and clinical trials.

## Introduction

In December 2019, a series of severe atypical respiratory disease cases occurred in Wuhan, China, and a novel coronavirus named severe acute respiratory syndrome coronavirus 2 (SARS-CoV-2) was rapidly identified as the causative agent of coronavirus disease 2019 (COVID-19). SARS-CoV-2 virions consist of a helical nucleocapsid formed by the association of nucleocapsid (N) phosphoproteins with viral genomic RNA that is surrounded by a lipid bilayer and three structural proteins, the spike (S), the membrane (M), and the envelope (E) proteins, are inserted into the nucleocapsid. The new coronavirus rapidly spread around the globe, resulting in the World Health Organization’s declaration of a pandemic on March 11, 2020. As of December 21, 2021, SARS-CoV-2 had caused over 275 million infections and more than 5.3 million deaths worldwide (Johns Hopkins University of Medicine Coronavirus Resource Center. COVID-19 dashboard). Although therapeutics including monoclonal antibodies and antivirals have shown some therapeutic efficacy in limiting the burden of COVID-19, effective vaccines are essential to control the pandemic. As of April 20, 2021, 13 vaccines had been approved by different health agencies worldwide, and over 10 vaccine candidates were being tested in phase 2/3 or phase 3 trials, including the plant-based vaccine candidate developed by Medicago tested in the present study.

The S glycoprotein contains a receptor-binding domain (RBD) that binds strongly to the human receptor angiotensin-converting enzyme 2, playing a major role in viral attachment, fusion, and entry into host cells [1, 2]. Neutralizing antibodies (NAbs) directed against the S protein are known to provide protection from other highly pathogenic coronaviruses (e.g., SARS-1 and Middle East respiratory syndrome (MERS)), and similar protective efficacy was rapidly demonstrated with anti-S antibodies in SARS-CoV-2 infection [3, 4]. Consequently, the S protein rapidly emerged as a prime target for the development of COVID-19 vaccines.

Medicago’s platform technology is based on transient expression of recombinant proteins in the nontransgenic plant *Nicotiana benthamiana*. *Agrobacterium tumefaciens* is used as a transfer vector to move targeted DNA constructs into plant cells. The newly introduced DNA then directs the expression of the desired recombinant proteins [5]. Plant-derived coronavirus-like particles (CoVLPs) are produced from the expression of a modified full-length S protein. In plant cells, newly synthesized S proteins trimerize and then move to lipid rafts in the plasma membrane, where they spontaneously assemble into virus-like particles (VLPs) that “bud” off the surface of the plant cell. The S proteins in a CoVLP are in a stabilized, prefusion conformation that resembles the native structure seen on SARS-CoV-2 virions. The prefusion form of the S protein is preferred as a vaccine antigen since it contains several epitopes in the RBD that are primary targets for NAbs [6]. Moreover, a previous study on the MERS S protein revealed that the prefusion state also resulted in a more potent immunogen with dose-sparing properties compared to a protein made with the original wild-type sequence [7]. In a pandemic situation, large numbers of vaccine doses are required to protect the maximum number of people worldwide. Dose-sparing adjuvants are often used to maximize the number of vaccine doses available, i.e., to reduce the amount of antigen needed to elicit a robust and persistent immune response [8]. Two adjuvants with demonstrated potential for dose sparing were included in the current study: the cytidine-phospho-guanosine (CpG)-containing immunostimulatory oligodeoxynucleotide sequence 1018 (CpG 1018: Dynavax) and α-tocopherol-containing oil-in-water emulsion Adjuvant System 03 (AS03: GSK).

Synthetic oligodeoxynucleotides such as CpG 1018 are Toll-like receptor 9 (TLR9) agonists that mimic the activity of naturally occurring CpG motifs found in bacterial DNA. CpG sequences are potent vaccine adjuvants that enhance immune responses in general and tend to promote T-helper type 1 (Th1)-type responses in particular. B cells and plasmacytoid dendritic cells are the main human immune cells that express TLR9. Activation of these cells by CpG DNA initiates an immunostimulatory cascade that culminates in the indirect maturation, differentiation, and proliferation of natural killer cells, T cells, and monocytes/macrophages that contribute to a Th1-biased immune response [9, 10]. CpG 1018 is the adjuvant used in the licensed hepatitis B vaccine HEPLISAV-B.

The AS03 initiates a transient innate immune response at the injection site and draining lymph node in animal models [11] and in human peripheral blood [12–14]. This innate immune response potentiates and shapes the adaptive immune response to the vaccine antigen, including both antibody and T cell responses, resulting in an increased response magnitude, breadth, durability, and antibody avidity [15–17]. AS03 has been used in the licensed pandemic A/H1N1pdm09 influenza vaccines Arepanrix H1N1 (in Canada), Q Pan H5N1 (in the USA), and Pandemrix (in Europe), of which 90 million doses have been administered worldwide, as well as in other licensed or candidate vaccines [18]. The present study assessed the safety, immunogenicity, and protective efficacy of two doses, administered 28 days apart, of a plant-produced VLP vaccine candidate for COVID-19 in rhesus macaques.

## Materials and methods

### Animal model

Male Indian rhesus macaques from 3.5 to 8 years old were sourced from the breeding colonies of the New Iberia Research Center (NIRC) of the University of Louisiana at Lafayette and distributed evenly into treatment groups based on their age and weight to assure comparable distribution across the treatments. All animals were negative for simian immunodeficiency virus, simian T cell leukemia virus, simian retrovirus, and herpes B virus. Animals were housed and maintained as per the National Institutes of Health guidelines at the NIRC in accordance with the rules and regulations of the Committee on the Care and Use of Laboratory Animal Resources. The entire study (protocol 2020-8721-007) was reviewed and approved by the Institutional Animal Care and Use Committee (IACUC). For the challenge protocol, animals were transferred to the Regional Biosafety Level 3 (BSL3) facility at the Tulane National Primate Research Center (TNPRC), where the study was reviewed and approved by the Tulane University IACUC (Protocol P0450). All animals were cared for in accordance with the Institute for Laboratory Animal Research Guide for the Care and Use of Laboratory Animals 8th Edition. The Tulane University Institutional Biosafety Committee approved the procedures for sample handling, inactivation, and removal from BSL3 containment.

### Vaccine and adjuvants

The CoVLP vaccine candidate expressing the full-length S glycoprotein of the SARS-CoV-2 strain hCoV-19/USA/CA2/2020 was produced as previously described [5, 19]. The S protein was modified with R667G, R668S, and R670S substitutions at the S1/S2 cleavage site to increase stability and K971P and V972P substitutions to stabilize the protein in the prefusion conformation [20]. Self-assembled VLPs bearing S protein trimers were isolated from the plant matrix and subsequently purified [5]. A 6-month stability approbation was requested from the relevant regulatory agencies. AS03 (provided by GSK, Rixensart, Belgium) is an o/w emulsion containing 11.86 mg dl-α-tocopherol, 10.69 mg squalene, and 4.86 mg polysorbate 80 per 250 µL, which corresponds to one human dose. CpG 1018, a synthetic 22-base phosphorothioate oligodeoxynucleotide containing an immunostimulatory CpG sequence in sterile Tris-buffered saline solution, was provided by Dynavax (Emeryville, CA, USA).

### Study design

**Fig. 1:**
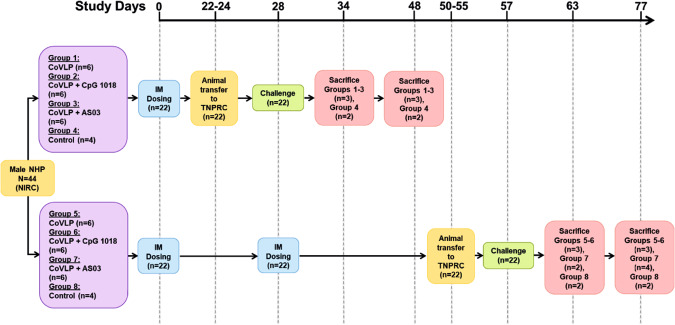
Experimental design. Male Indian rhesus macaques were immunized with one (Day 0) or two (Days 0 and 28) intramuscular (IM) doses of 15 µg of CoVLP (formulated or not with the adjuvant CpG 1018 or AS03) or control. Animal immunization and safety evaluation before challenge were performed at the New Iberia Research Center. Approximately 1 week before the challenge, the animals were transferred to the Tulane National Primate Research Center for the second part of the protocol, which included viral infection and safety and protection evaluations. Animals were challenged with SARS-CoV-2 USA-WA1/2020 on Day 28 (animals immunized with one dose) or Day 57 (animals immunized with two doses) and sacrificed 6 or 20 days after the challenge

Male Indian rhesus macaques received one or two doses of 15 µg in 0.5 mL/dose for the unadjuvanted CoVLP, 15 µg in 0.5 mL/dose for the AS03-adjuvanted CoVLP (1:1 ratio v/v), 15 µg in 1 mL/dose for the CpG 1018 (3 mg/dose)-adjuvanted CoVLP, or control phosphate-buffered solution (placebo). Adjuvanted vaccines were mixed immediately prior to dosing. Animals were immunized intramuscularly (IM) in the deltoid muscle with one (on Day 0) or two doses administered 28 days (on Day 28) apart. Approximately 1 week before the challenge, animals were transferred to the TNPRC for a viral challenge. Animals were infected (detailed below) with SARS-CoV-2 USA-WA1/2020 on Day 28 (one-dose groups) or Day 57 (two-dose groups) and euthanized 6 or 20 days after challenge (Fig. 1). The treatment groups are detailed in Supplementary Table 1. For logistic reasons, the study was carried out in two cohorts of four groups each (i.e., the one- and two-dose cohort).

### Prechallenge safety evaluation

Clinical observations, including general behavior and visible signs of disease (reduced appetite, hunched posture, pale appearance, and dehydration), were performed daily before the challenge.

Body temperature and weight were measured at each scheduled anesthetic event, i.e., on Day 0 (before immunization) and Days 1, 3, and 7 after the first immunization for all groups as well as on Day 28 (before the second immunization), Days 29, 32, and 35 for the two-dose cohort.

The injection sites were monitored for 7 days after each immunization using standard measures, including upper arm circumference, lymph node enlargement (size, mobility, and number), warmth, erythema, local lesions (ulcers and abscess), and other abnormalities. Each of these parameters was graded throughout the study by the same experienced investigator blinded to group assignment (0 = absent, 1 = mild, 2 = moderate, and 3 = severe). Upper arm circumference and lymph node enlargement were manually assessed at scheduled anesthetic events up to 7 days after each injection, i.e., Days 0, 1, 3, and 7, as well as Day 28 (before the second immunization), Days 29, 32, and 35 for the two-dose cohort. Routine hematological and biochemical analyses (detailed in Supplementary Tables 5–8) were performed on whole blood samples from all animals. Blood samples were collected before immunization (baseline) and 1, 3, and 7 days after each immunization.

### Anti-RBD IgG, IgA, and IgM

Anti-RBD immunoglobulin G (IgG), IgA, and IgM in the serum were measured by laboratory staff blinded to group allocation using an enzyme-linked immunoassay (ELISA) method adapted for nonhuman primate (NHP) serological testing from Rӧltgen and coauthors [21]. Briefly, 96-well high-binding ELISA plates were coated with a SARS-CoV-2 RBD protein at a concentration of 0.2 µg/well. Following washing and blocking steps, the wells were incubated with NHP serum samples at a dilution of 1:100. The dilution of 1:100 was determined through method optimization covering a 1:50 to 1:25,600 dilution range for sera from four uninfected and four SARS-CoV-2-infected NHPs. After washing to remove unbound antibodies, specific RBD-bound IgG, IgA, or IgM antibodies were detected by horseradish peroxidase-conjugated goat anti-monkey IgG (γ-chain-specific, Alpha Diagnostics Intl., San Antonio, TX), IgM (μ-chain-specific, Alpha Diagnostics Intl.), or IgA (α-chain-specific, Alpha Diagnostics Intl.) secondary antibodies. After washing, 3,3′,5,5′-tetramethylbenzidine substrate solution was added, and the reaction was stopped using sulfuric acid. The optical density at 450 nm was measured with an EMax Plus microplate reader (Molecular Devices, San Jose, CA, USA); values for blank wells were subtracted from values obtained for serum-containing samples. All samples were run in duplicate.

### NAb titers

NAb titers in serum samples were measured at baseline, Day 21, and Day 49 by laboratory staff blinded to group allocation at the Battelle Biomedical Research Center (Columbus, OH) using a SARS-CoV-2 microneutralization (MN) assay with an in situ ELISA readout against a SARS-CoV-2 N protein. Briefly, serum samples were incubated with the SARS-CoV-2 WA 1/2020 isolate at 37.0 °C and 5.0% CO_2_ for 1 h. The serum/virus mixture was then transferred to Vero E6 cell monolayers and incubated for 40 to 46 h. The plates were rinsed with Hank’s balanced salt solution and fixed with 80% acetone. Following fixation, an in situ ELISA was performed for the detection of the SARS-CoV-2 N protein antigen using an anti-SARS-CoV-2, MERS, SARS-CoV primary antibody (EastCoast Bio, North Berwick, ME), and a goat anti-mouse IgG-conjugated antibody (Fitzgerald, Acton, MA). Samples were analyzed in duplicate, and results are reported as the median of two independent analyses. The final value for each sample is expressed as the MN50 value calculated as the reciprocal of the serum dilution that neutralized ≥50% of SARS-CoV-2 WA 1/2020 virus. Nonresponders (MN50 value <20) were assigned a value of 10.

### Cell-mediated immune response

Frozen peripheral blood mononuclear cells (PBMCs) were thawed, counted, and resuspended at a density of 1 million live cells/mL in a complete RPMI medium (RPMI medium supplemented with 10% fetal bovine serum and antibiotics). Cells were rested overnight at 37 °C in a CO_2_ incubator. The next morning, the cells were counted again, resuspended at a density of 15 million/mL in complete RPMI medium, and 100 μL of cell suspension was added to each well of a 96-well round-bottomed tissue culture plate and stimulated ex vivo with a peptide pool consisting of 15-mer peptides overlapping by 11 amino acids spanning the S protein (GenScript, Piscataway, NJ) at a concentration of 1.2 μg/mL per peptide in the presence of 1 μg/mL anti-CD28. The cells were incubated at 37 °C in a 5% CO_2_ incubator for 2 h before the addition of 10 μg/mL Brefeldin-A (BioLegend, San Diego, CA). Four hours later, the PBMCs were washed with phosphate-buffered saline (PBS) and stained with Zombie UV fixable viability dye (BioLegend, San Diego, CA). The cells were washed with PBS containing 5% fetal calf serum before the addition of a surface antibody cocktail (Supplementary Table 2). The cells were stained for 20 min at 4 °C in a 100 μL volume. Subsequently, the cells were washed, fixed, and permeabilized with Cytofix/Cytoperm buffer (BD Biosciences) for 20 min. The permeabilized cells were stained with an intracellular cytokine staining antibody cocktail (Supplementary Table 2) for 20 min at room temperature in 1× perm/wash buffer (BD Biosciences). The cells were then washed twice with perm/wash buffer (BD Biosciences) and once with staining buffer before analysis using a BD Symphony flow cytometer. All flow cytometry data were analyzed using the FlowJo software v10 (TreeStar Inc., Ashland, OR).

### Challenge

Animals were infected under anesthesia with the viral isolate SARS-CoV-2 USA-WA1/2020 (originally obtained from a patient in Seattle, USA; kindly provided by the University of Texas, Medical Branch, Galveston, TX) as previously described [22, 23]. The virus stock was expanded on Vero E6 cells and titrated on that cell line by plaque assay. Deep sequencing showed no polymorphisms >5% of reads relative to the original isolate. The furin cleavage site, a site showing frequent culture adaptation in Vero E6 cells, harbored no polymorphisms >1% of sequence reads in this stock. Animals were challenged by multiroute administration, i.e., 500 µL intratracheal, intranasal (approximately 250 μL on each nare), and conjunctival (10 µL), with a total viral dose of 10^6^ plaque-forming units.

### Post challenge clinical observations and chest radiographs

Clinical observations, including state of responsiveness, presence of discharge, skin condition, respiratory condition, food consumption, and fecal conditions, were performed daily after challenge until termination by laboratory staff blinded to group allocation. Animals were individually scored for the severity of these parameters, and clinical scores were assigned according to the chart provided in the Supplementary Material (Additional information file S1). Thoracic radiographs were obtained on Days 6, 13, and 20 post challenge.

Body temperature, weight, respiratory rate, and oxygen saturation level (SpO_2_) were measured for all animals on the challenge day (before virus administration) and Days 6, 13, and 20 post challenge.

Routine hematologic and biochemical analyses (detailed in Supplementary Tables 5–8) were performed on whole-blood samples from all animals collected on Days 6, 13, and 20 post challenge.

### Viral replication in swabs and the BALF

Subgenomic RNA (sgRNA) levels of the *E* and/or *N* genes of SARS-CoV-2 in bronchoalveolar lavage fluid (BALF) and nasal and oropharyngeal swabs were measured by quantitative real-time polymerase chain reaction (RT-qPCR) on Days 6, 13, and 20 after challenge. Samples were collected in 200 µL of 1× DNA/RNA Shield (Zymo Research, Irvine, CA) and extracted for viral RNA (vRNA) using a Quick-RNA Viral Kit (Zymo Research). vRNA buffer was dispensed directly onto the swab in DNA/RNA Shield. A modification to the manufacturer’s protocol was used, which involved the insertion of the swab directly into the spin column for centrifugation, allowing all of the collected material to cross the spin column membrane. The extracted vRNA was then eluted (45 µL), and 5 µL was added in a 0.1-mL fast 96-well optical microtiter plate format (Thermo Fisher, CA) for a 20 µL RT-qPCR reaction volume. RT-qPCR was performed with TaqPath 1-Step Multiplex Master Mix (Thermo Fisher) along with the 2019-nCoV RUO Kit (IDTDNA, Coralville, IA), a premade mixture of forward and reverse primers, and a FAM-labeled probe targeting the N1 amplicon of the *N* gene or a leader sequence on the *E* gene of SARS2-nCoV19 (accession no.: MN908947). The RT-qPCR program was as follows: uracil-N-glycosylase incubation at 25 °C for 2 min, reverse transcriptase incubation at 50 °C for 15 min, and enzyme activation at 95 °C for 2 min, followed by 40 cycles of a denaturing step at 95 °C for 3 s and annealing at 60 °C for 30 s. Fluorescence signals were detected with an Applied Biosystems QuantStudio 6 Sequence Detector (Applied Biosystems, Foster City, CA). Data were captured and analyzed with Sequence Detector Software v1.3 (Applied Biosystems). The baseline was adjusted from 15 to 10 Cq based on the 10^8^ copy number of the calibration curve intersecting with the threshold line at 12 (±0.5) cycles. Viral copy numbers were determined by plotting Cq values obtained from unknown (i.e., test) samples against serial tenfold dilutions of exogenous calibration curves ranging from 10^1^ to 10^8^ log copy numbers generated from known amounts of in vitro-transcribed ssRNA for interpolation to determine the number of viral copies per mL of initial specimen sample or test dilution, as applicable. The limit of detection (LOD) of the assay was 10 copies per reaction volume. To control for assay variation, validation of results, internal positive control quantification consistency, and reagent stability, replicates of a positive control sample were also analyzed in parallel with every set of test samples. Negative control plasma samples were included for processing with every set of test samples to monitor cross-contamination. A nontemplate control was included in all runs to ensure that there was no cross-contamination between reactions.

### Chemokines and cytokines in the BALF

Cytokines were measured using 497 Mesoscale Discovery. Interleukin-16 (IL-16), IL-6, IL-10, IL-8, IL-2, IL-15, IL-12p40, C–C motif chemokine ligand 17 (CCL17), and monocyte chemoattractant protein-1 (MCP-1) were included in the V-PLEX Plus Proinflammatory Panel 1, V-PLEX Plus Chemokine Panel 1, and V-PLEX Plus Cytokine Panel 1 Human Kits (Meso Scale Diagnostics LLC Rockville, MD). The plate was read on a MESO Quickplex 500 SQ120 instrument. Values below the LOD were replaced with 50% of the LOD based on the lowest value of the standard curve for each analyte.

### Lung immunohistochemistry

Lung tissue sections were prepared as previously described [22]. Five-micrometer sections of formalin-fixed, paraffin-embedded lungs were incubated for 1 h with primary antibodies against SARS-CoV-2 N protein (mouse IgG1, catalog number 40143-MM08, Sino Biological, Wayne, PA), ionized calcium-binding adaptor (IBA1; rabbit polyclonal, catalog number 019-19741; Wako, Richmond, VA) or myeloperoxidase (MPO; rabbit polyclonal, catalog A0398, Agilent Dako, Santa Clara, CA) diluted in normal goat serum (NGS) at a concentration of 1:200, 1:100, and 1:6000, respectively. Secondary antibodies conjugated with fluorochromes and diluted 1:1000 in NGS were incubated for 40 min. Slides were imaged with a digital slide scanner (Zeiss Axio Scan. Z1, Zeiss, White Plains, NY). Quantification of stained cells was performed by laboratory staff who were blinded to treatment allocation.

### Statistical analysis

A repeated-measures two-way analysis of variance was performed on log-transformed values for IgA, IgM, IgG, and NAb data. Kruskal–Wallis tests followed by Dunn’s multiple comparisons post hoc analysis were conducted independently on cell-mediated immune (CMI) response data to identify significant differences among vaccine formulations or timepoints. Kruskal–Wallis tests followed by Dunn’s multiple comparisons post hoc analysis were also performed to identify significant differences in the cytokine and chemokine levels in the BALF among vaccine formulations. Differences were considered statistically significant if *p* < 0.05 (GraphPad Prism software version 8.4.2; GraphPad Software, La Jolla, CA).

## Results

### Prechallenge safety evaluation

None of the vaccine formulations induced any increase in body temperature during the 7 days after the first or second IM immunization. All animals, including those in the control group, lost weight during that study period after the first immunization, independent of treatment. No weight loss was observed after the second immunization. After the first dose, one animal in the CoVLP group and another in the CoVLP+AS03 group developed mild (grade 1) erythema at the injection site. Resorption of the erythema was observed within 2 to 3 days. No injection site observations were noted after the second dose.

After the first immunization, clinical observations were limited to reduced appetite (also seen in the control group) and loose or soft stool that occurred concurrently with normal stool. After the second immunization, no clinical signs were observed.

The hematology results did not reveal any safety concerns up to 7 days after the first or second immunization. CoVLP+AS03 administration led to the transient mobilization of immune cells in the periphery one day after the first and second doses. This was mainly manifested by increases in neutrophils and monocytes after the first immunization (Supplementary Fig. 1, left panel) and an increase in neutrophils after the second immunization (Supplementary Fig. 1, right panel). These hematological changes were accompanied by a transient C-reactive protein (CRP) increase, reflecting the development of an inflammatory reaction upon vaccination with the AS03-adjuvanted CoVLP (Supplementary Fig. 1, bottom panel). Immune cell mobilization and CRP increases had resolved by 3 days after immunization and were not observed with unadjuvanted CoVLP or CoVLP+CpG treatment (Supplementary Fig. 1).

### Humoral immune response

**Fig. 2:**
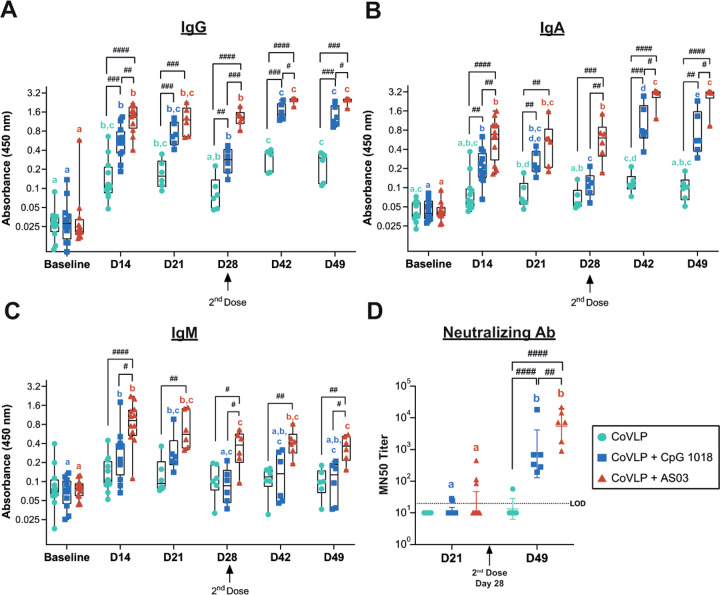
Humoral immune response. Anti-receptor-binding domain (RBD) (**A**) IgG, (**B**) IgA, (**C**) IgM, and (**D**) neutralizing antibody responses in the serum of macaques after the first and second immunizations, administered 28 days apart, with 15 µg of CoVLP with and without CpG 1018 or AS03. The geometric mean (GMT) and 95% CI are represented. The presence of identical color-matched letters indicates the absence of significant differences between two timepoints for each group (*p* < 0.05). Significant differences among groups at each timepoint are indicated by # (^#^*p* < 0.05, ^##^*p* < 0.01, ^###^*p* < 0.001, and ^####^*p* < 0.0001). Two-way ANOVA of log-transformed optical density (450 nm) values (GraphPad Prism v8, San Diego, USA)

RBD-specific IgG, IgA, and IgM were measured in preimmune samples and samples collected on Days 14, 21, or 28 after the first immunization as well as Days 14 and 21 after the second immunization (Day 42 and Day 49, respectively). The unadjuvanted CoVLP induced a significant increase in RBD-specific IgG as early as 14 days after the first immunization (Fig. 2A), which was maintained after 21 and 28 days. Both CpG and AS03 significantly increased RBD-specific IgG at all timepoints after immunization compared to the amount of RBD-specific IgG induced by the unadjuvanted CoVLP, with AS03 having a significantly greater impact than CpG. All groups benefited from booster immunization sustaining the higher responses of the adjuvanted formulations compared to the unadjuvanted CoVLP after the second dose (Fig. 2A). The superior response induced by CoVLP+AS03 was maintained at all timepoints after the booster immunization. Serum RBD-specific IgA responses were similar to RBD-specific IgG responses in intensity and ranking among the groups (Fig. 2B). Titers rose slightly in response to the unadjuvanted CoVLP, but barely reached statistical significance above baseline values after either the first or second dose. Animals that received an adjuvanted CoVLP mounted stronger IgA responses that rose significantly at Day 14 and further increased with the second dose. Again, the IgA responses were consistently highest in the CoVLP+AS03 group at all timepoints. Animals immunized with two doses of CoVLP+CpG experienced a small but significant decrease in RBD-specific IgA levels between Days 42 and 49 (Fig. 2B).

Although RBD-IgM levels remained significantly above baseline at all timepoints in the CoVLP+AS03 group, these levels tended to fall in all groups at later times after the first dose and were not greatly influenced by the booster dose (Fig. 2C), consistent with immunoglobulin class switching and the normal kinetics of IgM production following vaccination. RBD-specific IgM levels also rose in response to vaccination in the CoVLP+CpG group, but reached statistical significance only transiently at Days 14 and 21 (Fig. 2C).

Serum NAb titers were measured on Days 21 and 49. None of the animals in the CoVLP group mounted a detectable NAb response after the first dose, and NAbs were detected in only 1/6 of the animals after the second dose. Among the animals that received an adjuvanted vaccine, only 2/12 and 3/12 in the CoVLP+CpG and CoVLP+AS03 groups, respectively, mounted detectable responses 21 days after the first dose (Fig. 2D). The responses after the second dose were significantly above the baseline values in both the CoVLP+CpG and CoVLP+AS03 groups, illustrating the significant impacts of both adjuvants on the NAb response, although the levels in the CoVLP+AS03 group after the second dose were significantly higher than those in the CoVLP+CpG group.

### CMI response

**Fig. 3:**
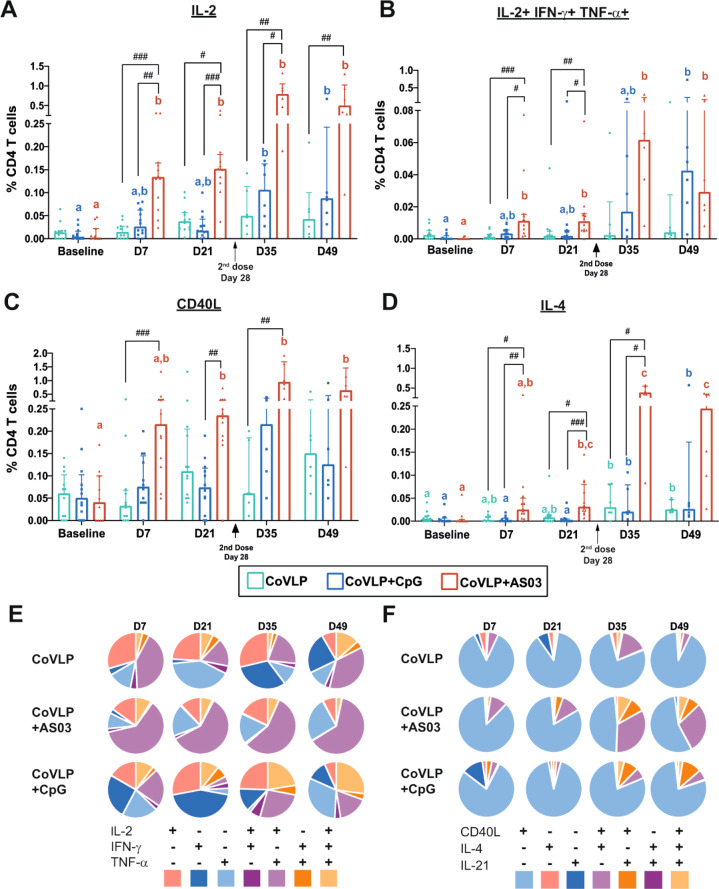
Cell-mediated immune response in PBMCs of macaques at baseline and 7 and 21 days after the first and second immunizations with 15  µg of CoVLP with and without CpG 1018 or AS03. (**A**) Percentages of S protein-specific IL-2+, (**B**) triple-positive IL-2+ IFN-γ+ TNF-α+, (**C**) CD40L+, and (**D**) IL-4+ CD4 T cells. The median and interquartile ranges are represented. Within each treatment group, significant differences among timepoints are annotated as letters, with the same letter on two bars indicating that no significant difference was detected between the two timepoints. Differences were considered statistically significant if *p* < 0.05. Significant differences among groups at each timepoint are indicated by # (^#^*p* < 0.05, ^##^*p* < 0.01, and ^###^*p* < 0.001). Kruskal–Wallis test with the Dunn post hoc multiple comparisons test (GraphPad Prism v8.4.2, San Diego, USA). Qualitative distributions of (**E**) Th1 and (**F**) Th2 CD4 T cells identified by the expression of IL-2/IFN-γ/TNF-α and CD40L/IL-4/IL-21, respectively

The CMI response was measured in PBMCs restimulated ex vivo with an S protein peptide pool at baseline and 7 and 21 days after the first and second IM immunizations (Days 7 and 21 for the first immunization and Days 35 and 49 for the second immunization). A single dose of 15 µg CoVLP+AS03 induced a significant increase in Ag-specific IL-2+ CD4 T cells in PBMCs as soon as 7 days after immunization with a clear boosting effect (Fig. 3A). Ag-specific IL-2+ CD4 T cells also significantly increased from baseline after the second dose of CoVLP+CpG. The higher response elicited by CoVLP+AS03 resulted in statistically significant differences between that group and both the unadjuvanted CoVLP and CoVLP+CpG groups at all timepoints, with the exception of the CoVLP+CpG group on Day 49. No significant differences in Ag-specific IL-2+ CD4 T cell responses were observed between the unadjuvanted CoVLP and CoVLP+CpG groups (Fig. 3A). The adjuvanted CoVLPs also increased the number of polyfunctional CD4 T cells, including triple-positive Th1 (IL-2+ IFN-γ+ TNF-α+) CD4 T cells (Fig. 3B). While this increase reached statistical significance only 21 days after the booster immunization (Day 49) in the CoVLP+CpG group, a significant increase from baseline was detected as soon as 7 days after the first dose in animals vaccinated with CoVLP+AS03. Significantly higher levels of triple-positive Th1 CD4 T cells compared to the baseline level were maintained in the AS03-adjuvanted group at all timepoints (Fig. 3B). However, due to the impact of the second dose of CoVLP+CpG on the quality of the CD4 T cell response (detailed below), similar levels of triple-positive Th1 CD4 T cells were observed for both adjuvanted formulations after the booster immunization. An increase in CD4 T cells expressing CD40L was also observed, particularly in the CoVLP+AS03 group (Fig. 3C), and most of the polyfunctional Th1 CD4 T cells, including the triple-positive CD4 T cells, expressed CD40L.

Significant induction of Ag-specific IL-4+ cells was observed after the booster immunization in all groups, particularly in the NHPs immunized with CoVLP+AS03 (Fig. 3D). However, the frequencies of Ag-specific IL-4+ CD4 T cells remained lower than those of Ag-specific IL-2+ CD4 T cells, even in the AS03-adjuvanted group.

Qualitative differences were observed in the CD4 T cell responses elicited by the different vaccine regimens (Fig. 3E, F). The response induced by CoVLP+AS03 was characterized by a higher frequency of IL-2+ TNF-α+ (IFN-γ−) CD4 T cells and remained relatively consistent with a minimal impact by the booster immunization on the relative proportions of subpopulations defined by the expression of Th1 cytokines (Fig. 3E).

In contrast, the quality of the Th1 response was strongly impacted by booster immunization in the unadjuvanted CoVLP and CoVLP+CpG groups, and the change was characterized by a predominance of monovalent CD4 T cells before the booster immunization and emergence of polyfunctionality after. Notably, the relative proportion of triple-positive IL-2+ IFN-γ+ TNF-α+ CD4 T cells increased after the second dose of CoVLP+CpG (Fig. 3E). The Ag-specific IL-4+ CD4 T cell response was strongly impacted by the second immunization with CoVLP+AS03, exhibiting the emergence of IL-4+ IL-21+ CD4 T cells (Fig. 3F). The Th2 responses in the CoVLP and CoVLP+CpG groups were characterized by the dominance of monovalent CD40L+ CD4 T cells (Fig. 3F).

### Post challenge clinical observations

In agreement with previously reported SARS-CoV-2 challenge in rhesus macaques, infection resulted in only mild symptoms in control animals [23, 24], with no significant differences in clinical score between groups (Supplementary Fig. 2). Animals in all groups experienced minor and transient reductions in peripheral oxygen saturation 6 days post challenge, and slightly increased breathing effort was noted in all groups, except the CoVLP+CpG group. Soft stool, accompanied by liquid stool on some occasions, was observed after challenge in all groups and resolved by 20 days post challenge, except in the unadjuvanted CoVLP group. Slight but nonsignificant increases in temperature in control animals were observed on Days 6 and 13 post challenge (Supplementary Tables 3 and 4). A slight decrease in red blood cell distribution width (RDW) and an increase in mean platelet volume (MPV) were noted in all treatment groups following infection. Among animals challenged after two doses of the CoVLP, slight increases in leukocyte and neutrophil counts were noted in all treatment groups (Supplementary Tables 5 and 6). On Day 6 post challenge, a transient increase in monocytes was observed in all experimental groups compared to the corresponding prechallenge levels. On Day 13 post challenge, monocyte counts were similar to those before the challenge. On Days 6 and 13 post challenge, an increase in the number of lymphocytes was noted for animals treated with the CoVLP alone or formulated with AS03. On Day 20 post challenge, the lymphocyte levels of all animals were similar to the corresponding prechallenge values (Supplementary Tables 5 and 6). Blood chemistry values appeared to reflect the impact of infection regardless of the treatment administered (Supplementary Tables 7 and 8).

### Post challenge thoracic radiographs

Thoracic radiographs were performed on Days 6, 13, and 20 post challenge. Not surprisingly, given the mild clinical course in the challenged animals, the radiographic changes and observations associated with infection were very subtle and limited.

*Among animals challenged after one dose*, no abnormal observations were reported on Day 6 after infection in animals immunized with the unadjuvanted CoVLP. Tracheobronchial lymph node enlargements were reported in one lung for 2/6 animals in the CoVLP+CpG group, and loss of definition in the right caudal lung field was reported for 1/6 of the animals in the CoVLP+AS03 group (Table 1). A subtle increase in pulmonary opacity was observed at all time points after infection in one animal vaccinated with CoVLP+AS03 (Table 1). The incidence of radiographic findings was slightly higher in the control group (1/4 of the animals at 6 days after infection, 2/2 of the animals at 13 days after infection, and 2/2 of the animals at 20 days after infection).

**Table 1:** Thoracic radiograph observations and incidence in rhesus macaques infected with SARS-CoV-2 after one or two immunizations with unadjuvanted CoVLP, CoVLP+CpG, or CoVLP+AS03

	Day 6								Day 13								Day 20							
	CoVLP		CoVLP+CpG		CoVLP+AS03		Control		CoVLP		CoVLP+CpG		CoVLP+AS03		Control		CoVLP		CoVLP+CpG		CoVLP+AS03		Control	
Lungs	R	L	R	L	R	L	R	L	R	L	R	L	R	L	R	L	R	L	R	L	R	L	R	L
Animals challenged after one dose																								
TBLN enlargement	0/6	0/6	**1**^**a**^**/6**	**1**^**a**^**/6**	0/6	0/6	0/4	0/4	0/3	0/3	0/3	0/3	0/3	0/3	0/2	0/2	0/3	0/3	0/3	0/3	0/3	0/3	0/2	0/2
Increase in opacity	0/6	0/6	0/6	0/6	**1/6**	0/6	**1/4**	**1/4**	0/3	0/3	0/3	0/3	**1/3**	**1/3**	**2/2**	**2/2**	0/3	0/3	0/3	0/3	**1/3**	**1/3**	**2/2**	**2/2**
Loss of definition	0/6	0/6	0/6	0/6	**1/6**	0/6	0/4	0/4	0/3	0/3	0/3	0/3	0/3	0/3	0/2	0/2	0/3	0/3	0/3	0/3	0/3	0/3	0/2	0/2
Animals challenged after two doses																								
TBLN enlargement	**1/6**	**1/6**	0/6	**1/6**	0/6	0/6	0/4	**1/4**	0/3	0/3	0/3	0/3	0/4	0/4	0/2	0/2	0/3	0/3	0/3	0/3	**1/4**	**1/4**	0/2	0/2
Increase in opacity	**1/6**	**1/6**	0/6	0/6	0/6	0/6	0/4	**2/4**	**1/3**	**1/3**	0/3	0/3	0/4	0/4	0/2	0/2	**1/3**	**1/3**	0/3	0/3	0/4	0/4	0/2	0/2
Loss of definition	0/6	0/6	0/6	0/6	0/6	0/6	0/4	**1/4**	**1/3**	**1/3**	0/3	0/3	0/4	0/4	0/2	0/2	**1/3**	**1/3**	0/3	0/3	0/4	0/4	0/2	0/2

Thoracic radiographs were obtained on Days 6, 13, and 20 post challenge for all animals, and tracheobronchial lymph node enlargement, increase in opacity, and loss of definition were evaluated in the right and left lungs.

*R* right, *L* left, *TBLN* tracheobronchial lymph node

Bold values indicate the occurence of radiographic findings

^a^Reported on the left and right lungs from different animals

*Among animals challenged after two doses*, tracheobronchial lymph node enlargement was observed in the CoVLP, CoVLP+CpG, and control groups (1/6, 1/6, and 1/4 animals, respectively) at 6 days post challenge and in 1/4 of the animals in the CoVLP+AS03 group at 20 days after challenge. An increase in pulmonary opacity was observed in one animal (in both lungs) in the CoVLP group at each timepoint (1/6, 1/3, and 1/3, respectively) and in 2/4 of the animals in the control group at 6 days after infection. Loss of lung definition was observed in 1/4 of the animals in the control group at 6 days post challenge and in 1/3 of the animals in the CoVLP group at 13 and 20 days after infection. At least one finding in one lung was observed in 37% (6 days post challenge), 50% (13 days post challenge), and 50% (20 days post challenge) of the unvaccinated control animals (Table 1). Radiographic findings were lower overall in the vaccinated groups: 17% (6 days post challenge), 33% (13 days post challenge), and 33% (20 days post challenge) in the CoVLP group. The rates of findings were even lower in animals that received two doses of an adjuvanted formulation, i.e., 17% (6 days post challenge), 0% (13 days post challenge), and 0% (20 days post challenge) in the CoVLP+CpG group and 0% (6 days post challenge), 0% (13 days post challenge), and 25% (20 days post challenge) in the CoVLP+AS03 group.

Although these observations were limited by the small number of animals in each group and the modest pathogenicity of SARS-CoV-2 in rhesus macaques, the lower occurrence of lung findings in the vaccinated NHPs after challenge suggests that two doses of the adjuvanted CoVLP may have provided a degree of protection in the lower respiratory tract. These observations are also notable for the absence of any suggestion of vaccine-enhanced disease.

### Viral replication in swabs and the BALF

**Fig. 4:**
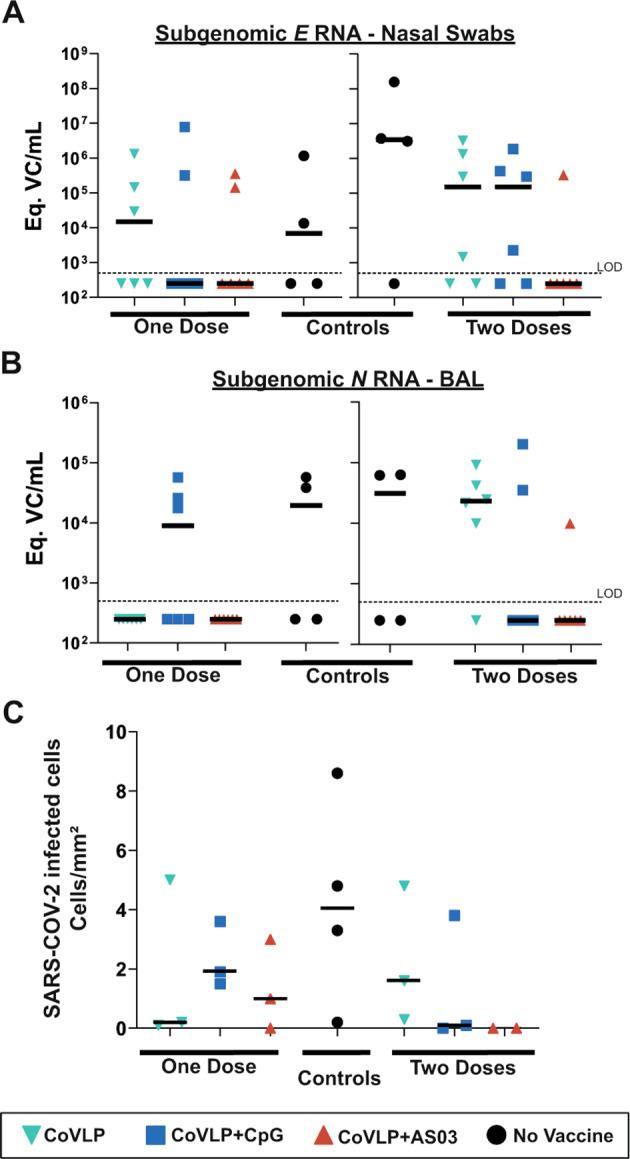
SARS-CoV-2 infection in the lungs at 6 days post challenge in rhesus macaques vaccinated with one or two dose(s) of CoVLP, CoVLP + CpG, or CoVLP + AS03. Equivalent (Eq.) viral copy (VC) of subgenomic *E* gene RNA in (**A**) nasal swabs and (**B**) subgenomic *N* gene RNA in the BALF were measured by real-time PCR. The dotted line represents the limit of detection of the method. Samples below the limit of detection were assigned a value of 250 Eq.VC/mL for graphical representation. (**C**) SARS-CoV-2-infected cells identified by immunohistochemistry and quantified. Individual data and the median (line) are represented

The viral loads in nasal swabs (sgRNA for the *E* gene) from eight control animals collected on Day 6 after the challenge were highly variable (range: undetectable to 10^8^ Eq.VC/mL). Although this variability and the relatively small number of animals in each treatment group limited our ability to perform statistical analyses, vaccination with one dose of the unadjuvanted CoVLP had no obvious effect on the viral load in nasal secretions, but a single dose of either adjuvanted CoVLP formulation appeared to reduce the median viral load by approximately 2 orders of magnitude, with undetectable sgRNA levels in nasal swabs in 8/12 (66.6%) animals compared to 2/4 (50%) in the control group (Fig. 4A). A two-log reduction in the nasal viral load was also observed in animals vaccinated with two doses of either the CoVLP alone or CoVLP+CpG compared to the control animals (Fig. 4A). An even greater 4-log reduction in the median sgRNA level was seen in NHPs vaccinated with two doses of CoVLP+AS03. In this group, sgRNA was detected in only 1/6 of the animals (16.6%) compared to 3/4 (75%) of the animals in the control group. No sgRNA was detected in pharyngeal swabs after two immunizations, except in a swab from one control animal. No sgRNA was detected in swab samples collected at 13 and 20 days post infection, with the exception of one animal that received two doses of the CoVLP alone. sgRNA (*E* gene) in the BALF was detected in only three vaccinated macaques at 6 days after challenge (one immunized with a single dose of CoVLP+CpG, one with two doses of CoVLP+CpG, and one with two doses of the CoVLP) and three control animals. A more sensitive PCR assay measuring the *N* gene product [25] was therefore used to measure viral replication in the BALF (Fig. 4B). In animals immunized with one dose, *N* sgRNA was not detected in animals treated with the CoVLP alone or CoVLP+AS03. Viral replication was observed in BALF samples from 3/6 (50%) and 2/4 (50%) of the animals in the CoVLP+CpG and control groups, respectively (Fig. 4B). In animals that received two doses, sgRNA was detected in only 1/6 (16.6%) of the animals in the CoVLP+AS03 group compared to 5/6 (83.3%) in the CoVLP alone group, 2/6 (33.3%) in the CoVLP+CpG group, and 2/4 in the control group (Fig. 4B). Neither *E* nor *N* gene sgRNA was detected in the BALF at 13 or 20 days after the challenge.

### SARS-CoV-2 infected cells and immune cell infiltration in the lungs

**Fig. 5:**
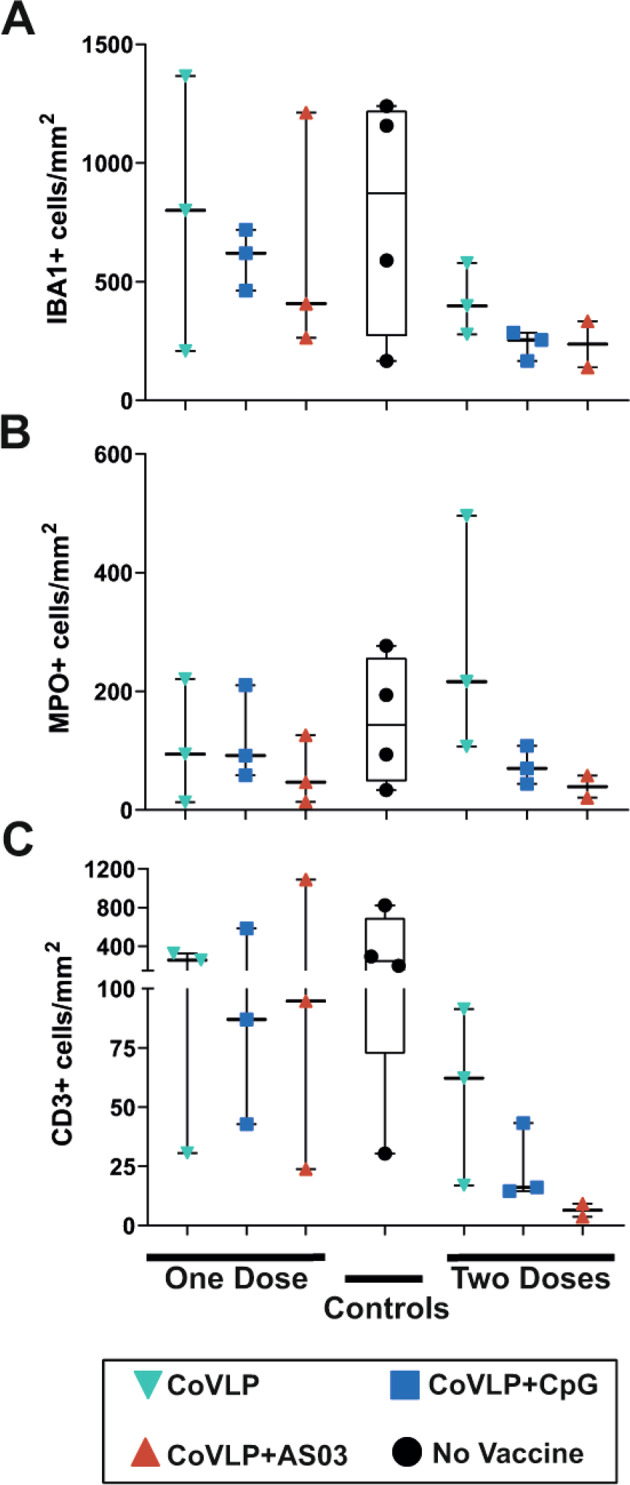
Immune cell infiltrations in lung sections at 6 days after challenge. (**A**) Macrophages (IBA1+ cells), (**B**) neutrophils (MPO+ cells), and (**C**) T lymphocytes (CD3+ cells) were quantified by immunohistochemistry. Individual data, medians (lines), and 75th/25th interquartiles (boxes) are represented. Results are expressed as positive cells/mm^2^ of tissue

SARS-CoV-2 infected cells and immune cell infiltration were detected by immunohistochemistry (Supplementary Fig. 3A, B) and quantified in lung sections on Day 6 post challenge (Fig. 4C). Fewer SARS-CoV-2 infected cells were observed in the lungs of most animals that had received at least one dose of the CoVLP with or without an adjuvant than in those of control animals, and no SARS-CoV-2 infected cells were detected in animals immunized with two doses of CoVLP+AS03 (Fig. 4C). The lungs of control animals were also characterized by the presence of immune cell infiltrates and expansion of the alveolar interstitium and perivascular spaces (Supplementary Fig. 3C, D). While vaccination with one dose of an adjuvanted CoVLP had a minor impact, immunization with two doses of the CoVLP strongly reduced the presence of macrophages, neutrophils, and T lymphocytes in the lungs at 6 days after infection (Fig. 5). Animals receiving two doses of an adjuvanted CoVLP had a 3-fold reduction in macrophages (Fig. 5A), a 2- to 4-fold reduction in neutrophils (Fig. 5B), and a 12- to 20-fold reduction in infiltrating T cells (Fig. 5C) compared to control animals.

### Chemokines and cytokines in the BALF after challenge

**Fig. 6:**
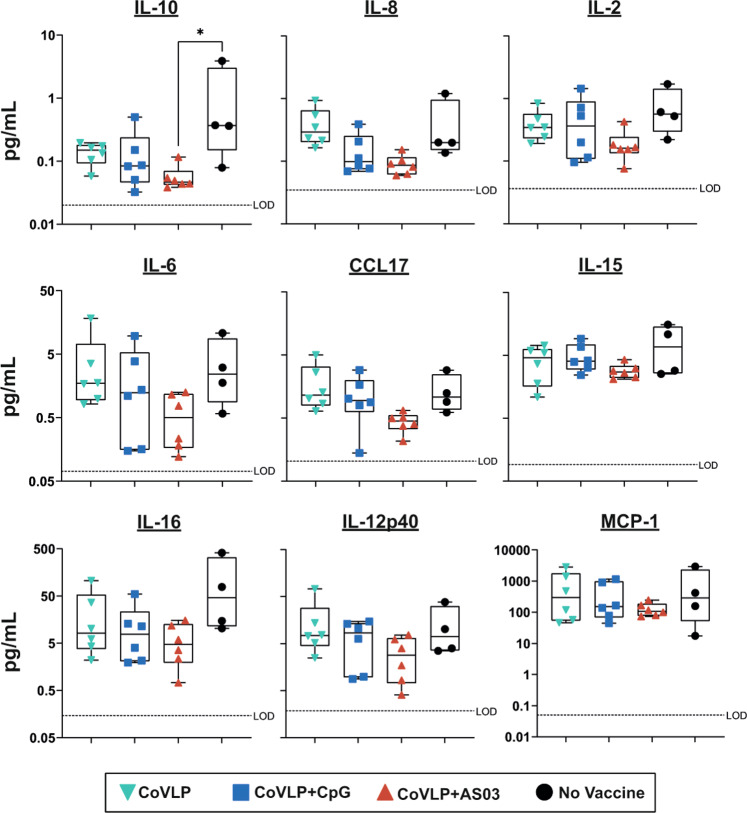
Cytokines and chemokines in BALF samples collected from rhesus macaques immunized with two doses of CoVLP, CoVLP + CpG, or CoVLP + AS03 on day 6 post challenge with SARS-CoV-2. Dotted lines indicate the limit of detection for each cytokine/chemokine. Individual data, medians (lines), and 75th/25th interquartiles (boxes) are represented. Statistical comparisons were performed using the nonparametric Kruskal–Wallis test followed by the Dunn post hoc multiple comparisons test (*p < 0.05)

The most pronounced differences in cytokine and chemokine profiles in the BALF between treatment groups were observed at 6 days post challenge in animals infected after administration of two vaccine doses (Fig. 6), while only limited differences were observed in animals challenged after one dose. In this context, it may be worth noting that the viral loads in nasal swabs from control animals in the two-dose cohort were generally higher than those from control animals in the one-dose cohort. Although levels varied widely among animals in most groups, the control animals tended to have the highest mean values for all of the cytokines and chemokines measured. Two doses of either the unadjuvanted CoVLP or CoVLP+CpG had relatively little impact on the cytokine and chemokine profiles, except for producing lower IL-10 and IL-16 levels than those in the control group (Fig. 6). Although these differences only reached significance for IL-10, the animals that received two doses of CoVLP+AS03 generally had consistently but marginally lower levels of most proinflammatory cytokines and chemotactic factors in the BALF than those in the control group (Fig. 6), a finding consistent with the radiologic and histopathologic evidence of reduced inflammation in the CoVLP+AS03 group.

## Discussion

No safety issues were observed after one or two injections of either the adjuvanted or unadjuvanted CoVLP vaccine candidate. The evidence of transient inflammation and mobilization of neutrophils (and to a lesser extent monocytes) in the blood of animals that received CoVLP+AS03 are consistent with the inflammatory response observed upon vaccination with AS03-adjuvanted vaccines [12–14].

Although one dose of the unadjuvanted CoVLP was sufficient to elicit anti-RBD IgG as soon as Day 14, both the addition of an adjuvant and administration of a second dose significantly improved the humoral response. The beneficial effects of CpG 1018 and AS03 on the humoral response have been previously demonstrated for hepatitis B [26–28] and influenza [16, 17] vaccines, respectively. More recently, the benefits of CpG 1018 (alone or with alum) or AS03 for the antibody response induced by COVID-19 vaccine candidates have been reported in NHPs and humans [19, 29, 30]. As observed in the present study, the vaccine candidates adjuvanted with AS03 elicited higher humoral and T cell responses than the CpG 1018-adjuvanted formulations. Monocyte-derived cells, rather than bona fide dendritic cells, seem to play an important role in the adjuvanticity of AS03. In addition to recruiting and activating cells at the site of injection, emulsions also favor the uptake of the antigen by antigen-presenting cells and subsequent transport to the draining lymph node. The presence of α-tocopherol has been associated with increased uptake of an antigen by monocytes, possibly contributing to the higher antibody response observed [11]. The IgG and IgA antibodies induced after the first dose appeared to have limited neutralizing potential, although moderate protection was observed in animals vaccinated with one dose of an adjuvanted CoVLP, illustrating the potential roles of non-NAbs and cell-mediated immunity [31–33]. The levels of NAbs induced after the second dose of CoVLP+AS03 have been reported to be protective in macaques following IgG adoptive transfer [34].

CoVLP+AS03 also elicited a strong IL-2-driven CD4 T cell response with a significant increase in polyfunctional cells, including triple-positive Ag-specific IL-2+ IFN-γ+ TNF-α+ Th1 CD4 T cells, as soon as 7 days after priming. Similar to the response induced by the AS03-adjuvanted inactivated split H5N1 influenza vaccine in humans [12, 17], the S protein-specific CD4 T cells induced by CoVLP+AS03 were characterized by the expression of CD40L. As expected, the CpG-adjuvanted formulation induced a more Th1-biased response characterized by a higher relative proportion of Ag-specific IFN-γ+ CD4 T cells compared to CoVLP+AS03. The response elicited by CoVLP+CpG was characterized by monovalent IL-2+ and IFN-γ+ CD4 T cells after the first dose and the emergence of polyfunctional CD4 T cells with minor induction of S protein-specific IL-4+ CD4 T cells after the second dose.

Human SARS-CoV-2 infection results in a Th1 response, and a large proportion of SARS-CoV-2-specific CD4+ T cells in the peripheral blood samples of recovered patients are directed against S protein epitopes [35, 36]. Studies of acute and convalescent COVID-19 patients have observed that T cell responses are associated with reduced disease [37, 38], suggesting that SARS-CoV-2-specific CD4+ and CD8+ T cell responses contribute significantly to infection control and resolution. Studies in mouse models have generally confirmed the critical role of T cells in viral clearance during coronavirus infections [39–41]. Moreover, polyfunctional Th1 cells that target viral antigens have been demonstrated to provide protection against other viral infections [42–44], supporting the hypothesis that S-specific CD4 T cells contribute to the observed reduction in the viral load in animals immunized with an adjuvanted CoVLP vaccine candidate. In addition, it was recently reported that the sequences of the vast majority of SARS-CoV-2 T cell epitopes, including epitopes in the S protein, are not affected by the mutations found in the predominant variants [45], in agreement with the potentially important role of the CMI response in cross-protection against emerging variants. The Th1 response elicited by CoVLP+AS03 was characterized by the predominance of IL-2+ TNF-α+ (IFN-γ−) CD4 T cells. Influenza-induced IL-2+ TNF-α+ (IFN-γ−) CD4 T cells have been described as uncommitted Th1-primed precursor (Thpp) cells preferentially induced by newly encountered antigens, whereas common influenza epitope-specific CD4 T cells induced by multiple booster immunizations express IFN-γ after infections [46, 47]. We previously reported the induction of Thpp cells after prime-boost immunizations (21 days apart) with VLPs presenting an H5N1 influenza hemagglutinin protein adjuvanted with either alum or glucopyranosyl lipid adjuvant-stable emulsion. Induction of Thpp CD4 T cells was also observed, although at a lower relative proportion, after prime-boost immunizations administered 21 days apart with a subunit vaccine consisting of the SARS-CoV-2 RBD displayed on a two-component protein nanoparticle adjuvanted with AS03 or alum [30]. Thpp cells have been proposed to serve as a reservoir of memory CD4+ T cells with effector potential [47]. As mentioned above, CoVLP+AS03 immunization was also associated with a higher humoral response. By secreting IL-4, Th2 cells can instruct B cells to produce IgG1 and IgE. The higher induction of CD40L+ IL-4+/IL-21+ cells observed in the CoVLP+AS03 group was consistent with the higher antibody response measured in these animals. Coexpression of CD40L and IL-21 can be indicative of T follicular helper (Tfh) cell engagement, which plays a critical role in B cell maturation and the establishment of memory [48, 49].

Although a relatively high viral dose was used for challenge experiments, the absence of major clinical signs is in agreement with previously reported challenge experiments in the rhesus macaque animal model [24, 30, 34, 50].

The levels of sgRNA in placebo control animals were higher or in agreement with previously reported values in NHPs after SARS-CoV-2 challenge [34, 50]. The reduced viral replication in nasal swabs, the lower incidence of clinical observations on thoracic radiographs, and the reduced number of SARS-CoV-2-infected cells in the lungs, especially in animals vaccinated with two doses of CoVLP+AS03, indicated the potential protection induced by the adjuvanted CoVLPs along with a reduction in viral shedding.

High levels of the proinflammatory cytokines IL-6 and IL-10 have been associated with severe outcomes during COVID-19 infection (e.g., hospitalization and intensive care unit admission) [51, 52], and low levels of CCL17 have been associated with lower viral replication in rhesus macaques, while high levels correlate with a higher viral load in bronchoalveolar brush samples [22]. Not surprisingly, the lower levels of proinflammatory cytokines and chemotactic factors were associated with less infiltration of immune cells in the lungs of animals immunized with two doses of an adjuvanted CoVLP. Although the migration of immune cells to the site of infection is essential to resolving a viral infection, the persistence of lymphoid cell infiltrates has been associated with uncontrolled inflammation and lung damage in coronavirus infections, including COVID-19 [53–56]. The nominal presence of immune cells associated with lower levels of proinflammatory cytokines and chemotactic factors in the BALF of vaccinated animals contributed to demonstrate the beneficial effect of two doses of an adjuvanted vaccine while concurrently demonstrating the absence of vaccine-associated enhanced disease.

The observed benefits provided by AS03 for CoVLP vaccination were consistent with reported increases in immunogenicity and protection [29, 30, 57]. The detailed CMI response reported in the present NHP study, including the possible engagement of Tfh cells, suggests a possible beneficial effect of AS03 on the durability of the immune response. Based on this study and the results from a phase 1 clinical trial [19], CoVLP+AS03 was selected for phase 2/3 studies.

## Supplementary information

Supplementary Figure 1

Supplementary Figure 2

Supplementary Figure 3

Supplementary tables

Scoring charts
