# Supplementary material for: Safety, immunogenicity, and protection provided by unadjuvanted and adjuvanted formulations of a recombinant plant-derived virus-like particle vaccine candidate for COVID-19 in nonhuman primates
Source: Cell Mol Immunol. 2022 Jan 5;19(2):222–33. doi: 10.1038/s41423-021-00809-2 (PMC8727235; doi:10.1038/s41423-021-00809-2)
Supplement: Supplementary file 1 — Supplementary Figure 1 [file 41423_2021_809_MOESM1_ESM.pdf]

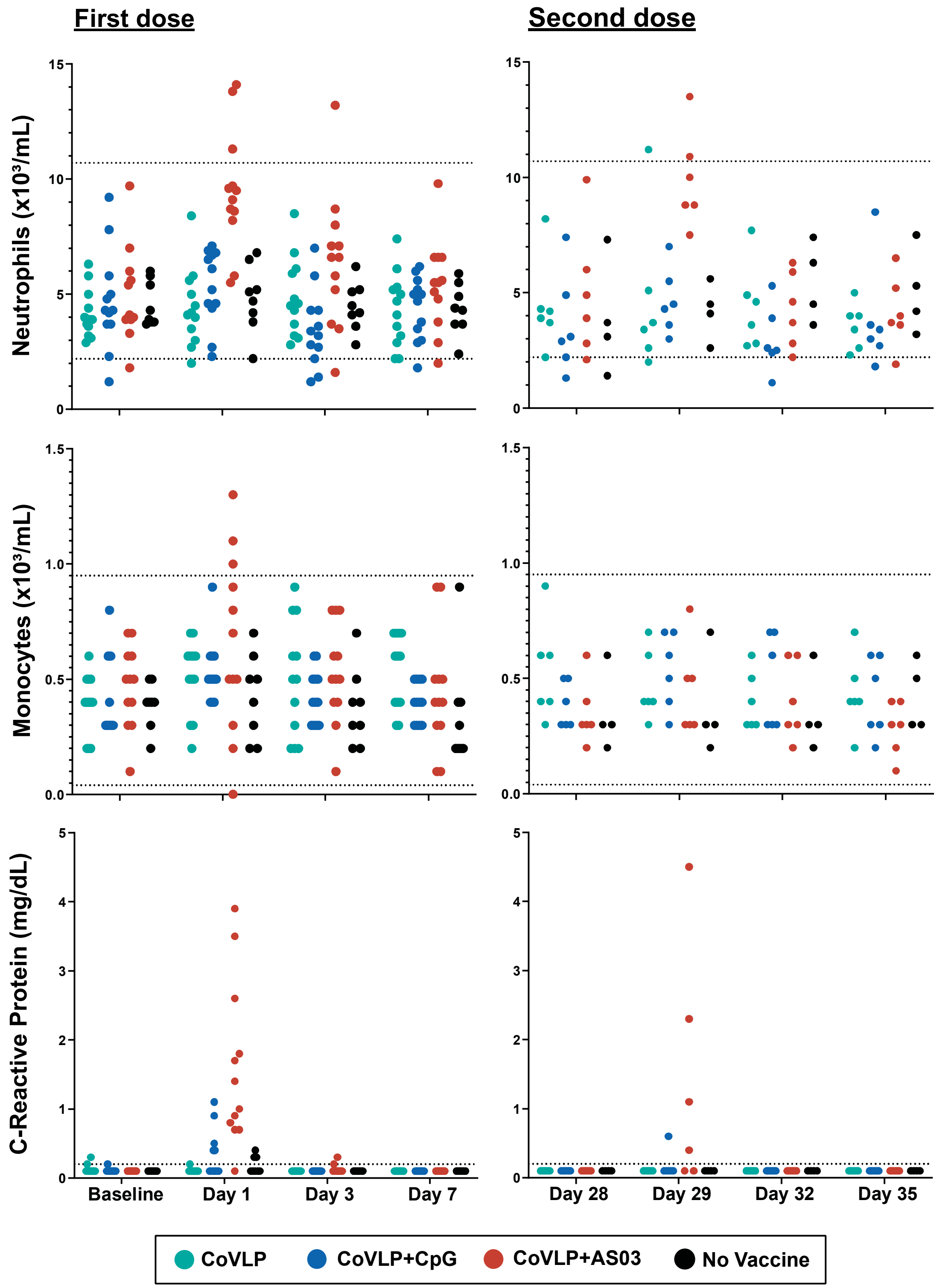

Supplementary Figure 1: Peripheral neutrophils, monocytes and serum C-reactive protein levels in rhesus macaques up to 7 days after the first (left panel) and the second (right panel) immunizations with CoVLP unadjuvanted or adjuvanted with AS03 or CpG 1018. Individual values are represented in each group. Dotted lines indicate limits of normal values.
