# Supplementary material for: Safety, immunogenicity, and protection provided by unadjuvanted and adjuvanted formulations of a recombinant plant-derived virus-like particle vaccine candidate for COVID-19 in nonhuman primates
Source: Cell Mol Immunol. 2022 Jan 5;19(2):222–33. doi: 10.1038/s41423-021-00809-2 (PMC8727235; doi:10.1038/s41423-021-00809-2)
Supplement: Supplementary file 2 — Supplementary Figure 2 [file 41423_2021_809_MOESM2_ESM.pdf]

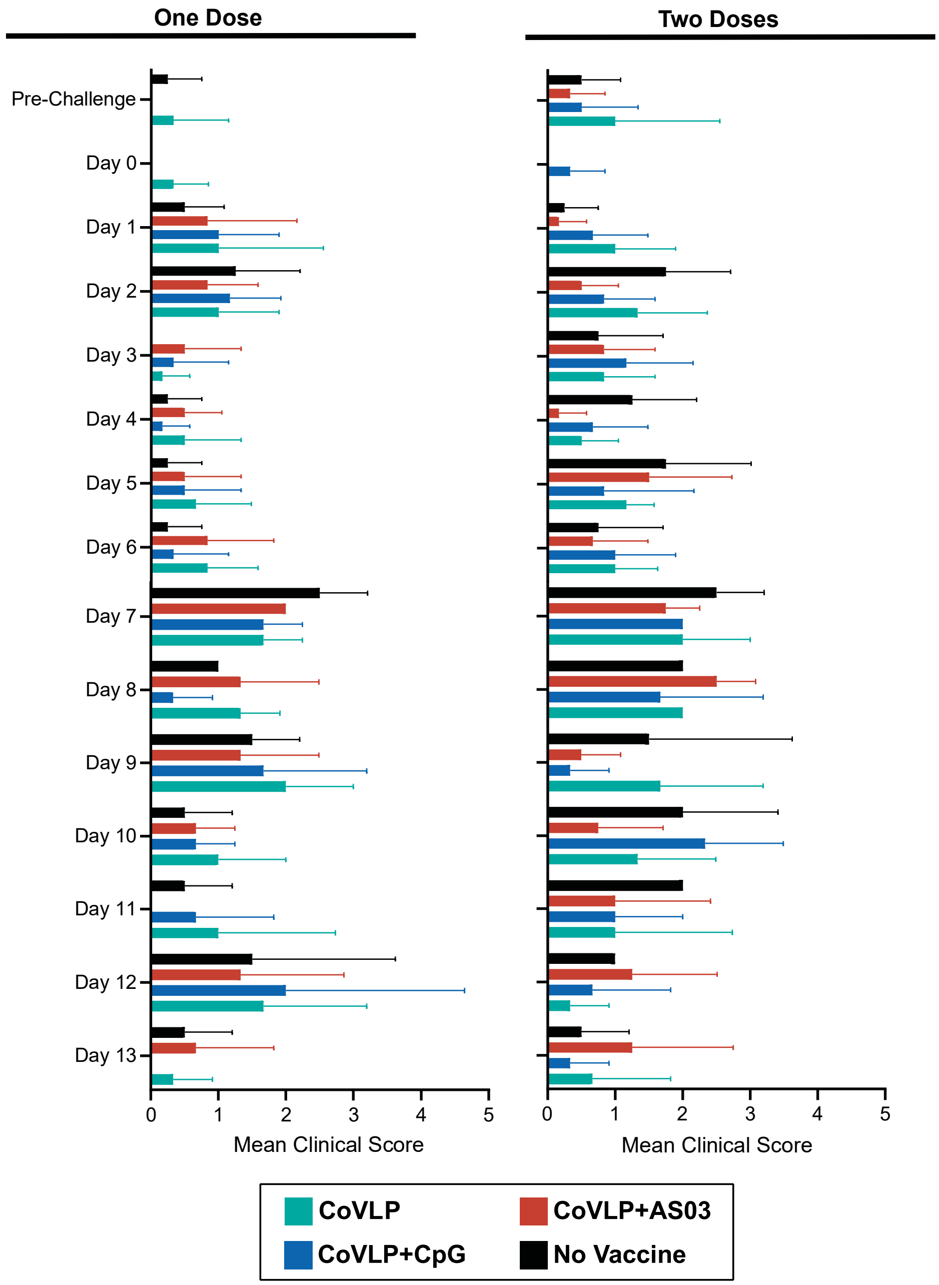

Supplementary Figure 2: Global clinical score of rhesus macaques infected with SARS-CoV-2 after challenge in animals immunized with one or two doses of CoVLP unadjuvanted or adjuvanted with AS03 or CpG 1018. Clinical observations were performed every day after challenge with SARS-CoV-2. Animals were individually scored for the severity of 6 different clinical observations (state of responsiveness, presence of discharge, skin condition, respiratory condition, food consumption and fecal condition). Global clinical score represents the addition of all clinical observations.
