# Supplementary material for: Safety, immunogenicity, and protection provided by unadjuvanted and adjuvanted formulations of a recombinant plant-derived virus-like particle vaccine candidate for COVID-19 in nonhuman primates
Source: Cell Mol Immunol. 2022 Jan 5;19(2):222–33. doi: 10.1038/s41423-021-00809-2 (PMC8727235; doi:10.1038/s41423-021-00809-2)
Supplement: Supplementary file 3 — Supplementary Figure 3 [file 41423_2021_809_MOESM3_ESM.pdf]

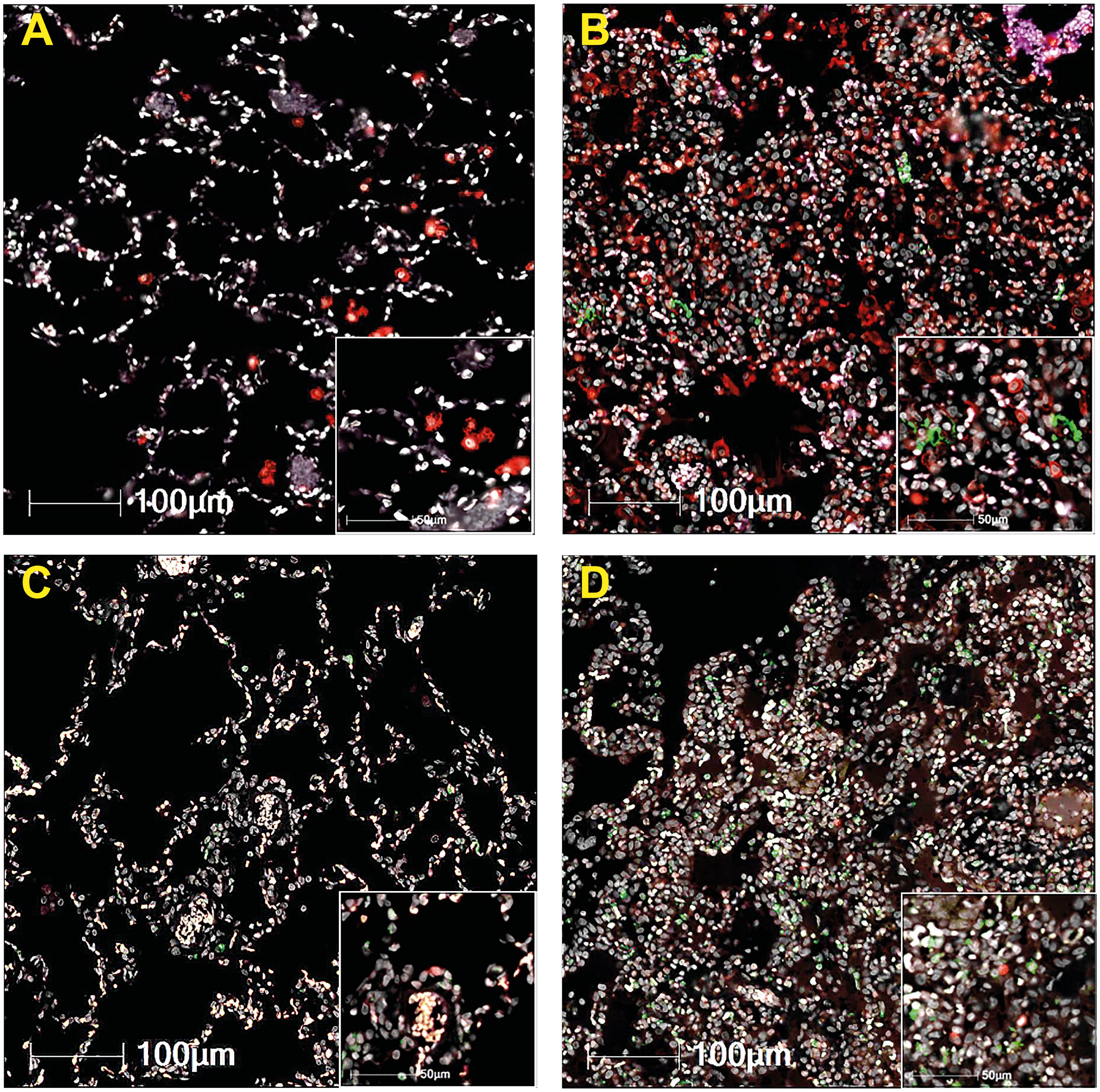

Supplementary Figure 3: Fluorescent immunohistochemistry of the lung from SARS-CoV-2 infected rhesus macaques 6 days post-challenge. Lung sections were stained for detection of macrophages (IBA1+, red) and SARS-CoV-2 nucleoprotein (green) (A and B) or T lymphocytes (CD3+, red) and neutrophils (MPO+, green) (C and D) in an animal with no detectable viral replication (A&C) or with high (>60,000 Eq. VC/mL) viral replication (B&D) in BAL.
