# Supplementary material for: Safety, immunogenicity, and protection provided by unadjuvanted and adjuvanted formulations of a recombinant plant-derived virus-like particle vaccine candidate for COVID-19 in nonhuman primates
Source: Cell Mol Immunol. 2022 Jan 5;19(2):222–33. doi: 10.1038/s41423-021-00809-2 (PMC8727235; doi:10.1038/s41423-021-00809-2)
Supplement: Supplementary file 4 — Supplementary tables [file 41423_2021_809_MOESM4_ESM.docx]

#### **Supplementary Table 1: Treatment groups**

| **Group No.** | **n** | **Group Description** | **CoVLP Dose (µg)^1^** | **Adjuvant Dose** | **Immunization (Days)** | **Challenge (Days)** |
| --- | --- | --- | --- | --- | --- | --- |
| 1 | 6 | CoVLP | 15 | 0 | 0 | 28 |
| 2 | 6 | CoVLP+CpG 1018 | 15 | 3 mg | 0 | 28 |
| 3 | 6 | CoVLP+AS03 | 15 | Ratio 1:1 (v:v) | 0 | 28 |
| 4 | 4 | Control (Cohort one dose) | -^(2)^ | 0 | 0 | 28 |
| 5 | 6 | CoVLP | 15 | 0 | 0 and 28 | 57 |
| 6 | 6 | CoVLP + CpG 1018 | 15 | 3 mg | 0 and 28 | 57 |
| 7 | 6 | CoVLP + AS03 | 15 | Ratio 1:1 (v:v) | 0 and 28 | 57 |
| 8 | 4 | Control (Cohort two dose) | -^(2)^ | 0 | 0 and 28 | 57 |
| ^1^ Total CoVLP proteins corrected by purity. ^2^ The no vaccine control animals were administered Phosphate Buffered Saline (PBS) Solution. | | | | | | |

#### **Supplementary Table 2: Immunological markers used to assess the cell-mediated response by flow cytometry**

|  |  | **Fluorochrome** | **Clone** | **Manufacturer** |
| --- | --- | --- | --- | --- |
|  | Viability | Zombie UV fixable viability dye | | BioLegend |
| Surface Stain | CD3 | BV650 | SP34-2 | BD Biosciences |
|  | CD4 | BV605 | OKT4 | BioLegend |
|  | CD8 | BUV563 | RPA-T8 | BD Biosciences |
| Intracellular Stain | CD154/CD40L | BV421 | 24-31 | BioLegend |
|  | IL-2 | FITC | MQ1-17H12 | BioLegend |
|  | IFN-γ | A700 | 4S.B3 | BioLegend |
|  | TNF-α | PE-Cy7 | Mab11 | E-Bioscience |
|  | IL-4 | PE | MP4-25D2 | BioLegend |
|  | IL-21 | APC | 3A3-N2 | BioLegend |

#### **Supplementary Table 3: Clinical signs in rhesus macaques infected with SARS-CoV-2 after one immunization with CoVLP unadjuvanted or adjuvanted with AS03 or CpG 1018**

| **Parameter** | | **Pre-Challenge^1^** | | | | **Post-Challenge^2^** | | | | | | | | | | | |
| --- | --- | --- | --- | --- | --- | --- | --- | --- | --- | --- | --- | --- | --- | --- | --- | --- | --- |
|  |  |  |  |  |  | **Day 6** | | | | **Day 13** | | | | **Day 20** | | | |
|  |  | **CoVLP** | **CoVLP + CpG  1018** | **CoVLP + AS03** | **Control** | **CoVLP** | **CoVLP + CpG 1018** | **CoVLP + AS03** | **Control** | **CoVLP** | **CoVLP + CpG 1018** | **CoVLP + AS03** | **Control** | **CoVLP** | **CoVLP + CpG 1018** | **CoVLP + AS03** | **Control** |
| Temp. Change (°C)^3,4^ | | 0.0±0.0 | 0.0±0.0 | 0.0±0.0 | 0.0±0.0 | 0.1±0.7 | 0.3±0.6 | 0.0±0.4 | 0.8±0.8 | 0.0±0.4 | 0.0±0.3 | 0.0±0.7 | 0.9±0.3 | -0.7±0.7 | -0.2±0.5 | -0.2±0.3 | 0.3±0.1 |
| 𝛿 Weight (%)^3,4^ | | 100±0 | 100±0 | 100±0 | 100±0 | 102±3 | 103±3 | 103±2 | 102±2 | 101±1 | 103±2 | 103±1 | 106±0 | 104±3 | 107±2 | 103±3 | 109±1 |
| Respiratory Rate^3^ | | 36±8 | 40±11 | 38±11 | 44±5 | 37±9 | 43±10 | 38±6 | 45±15 | 35±14 | 41±13 | 41±13 | 46±14 | 35±15 | 34±9 | 39±5 | 52±8 |
| SpO_2_ Change (%)^3,4^ | | 100±0 | 100±0 | 100±0 | 100±0 | 97±3 | 98±1 | 99±1 | 97±2 | 100±2 | 99±3 | 99±2 | 100+1 | 100±3 | 99±2 | 97±3 | 99±2 |
| Clinical Observations | Responsiveness (#) | 0/6 | 0/6 | 0/6 | 0/4 | 0/6 | 0/6 | 0/6 | 0/4 | 0/3 | 0/3 | 0/3 | 0/2 | 0/3 | 0/3 | 0/3 | 0/2 |
|  | Discharges (#) | 0/6 | 0/6 | 0/6 | 0/4 | 0/6 | 0/6 | 0/6 | 0/4 | 0/3 | 0/3 | 0/3 | 0/2 | 0/3 | 0/3 | 0/3 | 0/2 |
|  | Skin (#) | 0/6 | 0/6 | 0/6 | 0/4 | 0/6 | 0/6 | 0/6 | 0/4 | 0/3 | 0/3 | 0/3 | 0/2 | 0/3 | 0/3 | 0/3 | 0/2 |
|  | Increased Effort in Breathing (#) | 0/6 | 0/6 | 0/6 | 0/4 | **2/6** | **1/6** | **4/6** | **1/4** | **2/3** | **2/3** | **2/3** | **2/2** | **1/3** | 0/3 | **2/3** | **2/2** |
|  | Reduced Food Consumption (#) | 0/6 | 0/6 | 0/6 | **1/4** | **5/6** | **5/6** | **5/6** | **3/4** | **3/3** | **2/3** | **3/3** | **2/2** | **3/3** | **3/3** | **3/3** | **2/2** |
|  | Soft Stool (#) | **2/6** | 0/6 | 0/6 | 0/4 | **3/6** | **2/6** | **1/6** | **1/4** | **3/3** | **2/3** | **2/3** | **1/2** | **1/3** | 0/3 | 0/3 | 0/2 |
|  | Liquid Stool (#) | **1/6** | 0/6 | 0/6 | 0/4 | **1/6** | 0/6 | 0/6 | 0/4 | **1/3** | **1/3** | 0/3 | 0/2 | 0/3 | 0/3 | 0/3 | 0/2 |
| n=6 for CoVLP ± adjuvant groups and n=4 for control group at Day 0 pre-challenge and Day 6 post-challenge. n=3 for CoVLP ± adjuvant groups and n=2 at Days 13 and 20 post challenge. 𝛿: Delta (Change in) SD: Standard Deviation #: Occurrence of observations ^1^ Pre-Challenge represents data obtained before viral administration on the day of challenge, except for clinical observations for which observations were recorded one day before the challenge. ^2^ Clinical signs were observed every day. Day 6 data indicates the incidence observed between Days 0 to 6; Day 13 data indicates the incidence observed between Days 7 to 13; and Day 20 indicates the incidence observed between Days 14 to 20. ^3^ Data are presented as mean ± Standard Deviation. ^4^ Changes in temperature, weight and SpO_2_ are relative to challenge day (indicated as Pre-Challenge). | | | | | | | | | | | | | | | | | |

#### **Supplementary Table 4: Clinical signs in rhesus macaques infected with SARS-CoV-2 after two immunizations with CoVLP unadjuvanted or adjuvanted with AS03 or CpG 1018**

| **Parameter** | | **Pre-Challenge^1^** | | | | **Post-Challenge^2^** | | | | | | | | | | | |
| --- | --- | --- | --- | --- | --- | --- | --- | --- | --- | --- | --- | --- | --- | --- | --- | --- | --- |
|  |  |  |  |  |  | **Day 6** | | | | **Day 13** | | | | **Day 20** | | | |
|  |  | **CoVLP** | **CoVLP + CpG  1018** | **CoVLP + AS03** | **Control** | **CoVLP** | **CoVLP + CpG 1018** | **CoVLP + AS03** | **Control** | **CoVLP** | **CoVLP + CpG 1018** | **CoVLP + AS03** | **Control** | **CoVLP** | **CoVLP + CpG 1018** | **CoVLP + AS03** | **Control** |
| Temp. Change (°C)^3,4^ | | 0.0±0.0 | 0.0±0.0 | 0.0±0.0 | 0.0±0.0 | 0.4±0.3 | 0.2±0.5 | 0.4±0.3 | 0.4±1.0 | 0.3±0.5 | 0.1±0.8 | 0.3±0.5 | 0.6±0.4 | 0.1±0.3 | 0.3±0.8 | 0.4±0.6 | 0.0±0.7 |
| 𝛿 Weight (%)^3,4^ | | 100±0 | 100±0 | 100±0 | 100±0 | 103±3 | 103±3 | 103±2 | 102±1 | 102±2 | 104±3 | 103±3 | 102±1 | 105±2 | 111±2 | 109±5 | 107±1 |
| Respiratory Rate^3^ | | 31±4 | 31±9 | 30±5 | 37±8 | 32±7 | 38±13 | 28±5 | 42±15 | 31±6 | 28±4 | 28±5 | 40±11 | 44±7 | 45±9 | 40±7 | 45±30 |
| SpO_2_ Change (%)^3,4^ | | 100±0 | 100±0 | 100±0 | 100±0 | 100±4 | 98±2 | 98±2 | 97±2 | 100±1 | 97±1 | 101±1 | 97±1 | 100±2 | 99±2 | 101±2 | 101±2 |
| Clinical Observations | Responsiveness (#) | 0/6 | 0/6 | 0/6 | 0/4 | 0/6 | 0/6 | 0/6 | 0/4 | 0/6 | 0/4 | 0/6 | 0/2 | 0/3 | 0/3 | 0/4 | 0/2 |
|  | Discharges (#) | 0/6 | 0/6 | 0/6 | 0/4 | 0/6 | 0/6 | 0/6 | 0/4 | 0/6 | 0/4 | 0/6 | 0/2 | 0/3 | 0/3 | 0/4 | 0/2 |
|  | Skin (#) | 0/6 | 0/6 | 0/6 | 0/4 | 0/6 | 0/6 | 0/6 | 0/4 | 0/6 | 0/4 | 0/6 | 0/2 | 0/3 | 0/3 | 0/4 | 0/2 |
|  | Increased Effort in Breathing (#) | 0/6 | 0/6 | 0/6 | 0/4 | **2/6** | **2/6** | **5/6** | **2/4** | **2/3** | **2/3** | **4/4** | **1/2** | **2/3** | **3/3** | **4/4** | **1/2** |
|  | Reduced Food Consumption (#) | **1/6** | **1/6** | **2/6** | **1/4** | **6/6** | **6/6** | **5/6** | **4/4** | **3/3** | **3/3** | **4/4** | **2/2** | **2/3** | **3/3** | **4/4** | **2/2** |
|  | Soft Stool (#) | **1/6** | **1/6** | 0/6 | **1/4** | **2/6** | **2/6** | **1/6** | 0/4 | **2/3** | **1/3** | 0/4 | **1/2** | **1/3** | **1/3** | **3/4** | **1/2** |
|  | Liquid Stool (#) | **1/6** | 0/6 | 0/6 | 0/4 | 0/6 | 0/6 | 0/6 | 0/4 | 0/3 | 0/3 | **1/4** | 0/2 | 0/3 | 0/3 | 0/4 | 0/2 |
| n=6 for CoVLP ± adjuvant groups and n=4 for control group at Day 0 pre-challenge and Day 6 post-challenge. n=3 for CoVLP ± CpG 1018, n=4 for CoVLP + AS03 group and n=2 for Control group at Days 13 and 20 post challenge. Due to logistic reasons, animals were euthanized on Days 20, 21 or 23, results are presented as Day 20. 𝛿: Delta (Change in). #: Occurrence of observations. ^1^ Pre-Challenge represents data obtained before viral administration on the day of challenge, except for clinical observations for which observations were recorded one day before the challenge. ^2^ Clinical signs were observed every day. Day 6 data indicates the incidence observed between Days 0 to 6; Day 13 data indicates the incidence observed between Days 7 to 13; and Day 20 indicates the incidence observed between Days 14 to 20. ^3^ Data are presented as mean ± Standard Deviation. ^4^ Changes in temperature, weight and SpO_2_ are relative to challenge day (indicated as Pre-Challenge). | | | | | | | | | | | | | | | | | |

#### **Supplementary Table 5:** **Peripheral hematology in rhesus macaques infected with SARS-CoV-2 after one immunization with CoVLP vaccine, unadjuvanted or adjuvanted with AS03 or CpG 1018**

| **Parameter**  **(Normal values)** | **Pre-challenge** | | | | **Post-Challenge** | | | | | | | | | | | |
| --- | --- | --- | --- | --- | --- | --- | --- | --- | --- | --- | --- | --- | --- | --- | --- | --- |
|  |  |  |  |  | **Day 6** | | | | **Day 13** | | | | **Day 20** | | | |
|  | **CoVLP** | **CoVLP + CpG 1018** | **CoVLP + AS03** | **Control** | **CoVLP** | **CoVLP + CpG 1018** | **CoVLP + AS03** | **Control** | **CoVLP** | **CoVLP + CpG 1018** | **CoVLP + AS03** | **Control** | **CoVLP** | **CoVLP + CpG 1018** | **CoVLP + AS03** | **Control** |
| WBC (10^3^/µL)  (6.60-15.5) | 8.1±2.4 | 8.0±1.8 | 8.9±2.8 | 7.4±1.2 | 8.3±3.4 | 7.1±1.9 | 8.9±2.9 | 8.2±1.9 | 6.7±1.3 | 8.8±0.8 | 8.9±2.0 | 9.3±1.1 | 6.8±2.3 | 7.4±1.0 | 10.4±2.5 | 8.8±3.5 |
| RBC (10^6^/µL)  (4.1-7.8) | 5.11±0.43 | 5.33±0.39 | 5.38±0.34 | 5.53±0.32 | 5.39±0.19 | 5.34±0.33 | 5.42±0.22 | 5.54±0.26 | 5.53±0.24 | 5.31±0.36 | 5.47±0.21 | 5.28±0.52 | 5.61±0.34 | 5.27±0.29 | 5.44±0.20 | 5.45±0.07 |
| HGB (gm/dL)  (10.1-15.9) | 11.7±0.5 | 12.2±0.6 | 12.0±0.6 | 12.7±1.1 | 12.2±0.4 | 12.5±0.6 | 12.3±0.8 | 12.2±0.6 | 12.5±0.4 | 12.5±0.6 | 12.6±0.2 | 11.5±0.1 | 12.7±0.6 | 12.4±0.5 | 12.6±0.2 | 12.1±1.1 |
| Hematocrit (%)  (34.8-55.2) | 36.5±1.7 | 38.3±2.0 | 37.9±2.0 | 39.2±3.5 | 37.4±1.2 | 37.7±2.5 | 38.0±2.4 | 37.3±0.9 | 38.1±1.6 | 37.2± 2.2 | 38.4±0.7 | 35.3±1.4 | 39.0±2.2 | 37.1± 2.1 | 38.1±0.5 | 37.1±2.0 |
| MCV (fL)  (63.7-86.9) | 71.7±3.3 | 71.9±2.1 | 70.5±2.0 | 70.9±3.9 | 69.5±2.1 | 70.6±1.3 | 70.1±2.8 | 67.5±3.3 | 69.0±0.3 | 70.0±1.5 | 70.3±2.4 | 67.2±3.9 | 69.5±05 | 70.4±1.2 | 70.0±1.6 | 68.1±4.5 |
| MCH (pg)  (19.1-27.7) | 22.9±1.1 | 23.0±0.9 | 22.4±0.7 | 23.0±1.3 | 22.7±0.9 | 23.4±0.6 | 22.8±0.8 | 22.1±1.8 | 22.6± 0.4 | 23.7±1.3 | 23.1±0.8 | 21.9±2.4 | 22.7±0.4 | 23.6±1.0 | 23.1±0.8 | 22.1±2.3 |
| MCHC (gm/dL)  (28.9-35.4) | 32.0±0.3 | 31.9±0.5 | 31.8±0.3 | 32.5±0.4 | 32.6±0.4 | 33.1±0.6 | 32.5±0.3 | 32.8±1.0 | 32.8±0.4 | 33.7± 1.1 | 32.9±0.0 | 32.6±1.7 | 32.7±0.3 | 33.5±1.0 | 33.1±0.5 | 32.5±1.1 |
| RDW (%)  (10.9-15.3) | 14.4±0.8 | 14.1±0.9 | 14.0±0.5 | 13.9±0.7 | 12.6±1.0 | 12.3±1.0 | 12.1±0.2 | 13.9±0.5 | 12.2±1.1 | 12.2±0.7 | 12.0±0.1 | 14.4± 0.1 | 12.3±1.1 | 12.1±0.9 | 11.7±0.2 | 14.3±0.1 |
| Platelet (10^3^/µL)  (193.1-676.2) | 362±54 | 418±68 | 406±60 | 399±45 | 351±136 | 442±95 | 308±95 | 373±138 | 289±105 | 343±52 | 377±171 | 289±134 | 345±103 | 436±60 | 404±121 | 528±25 |
| MPV (fL)  (7.0-12.0) | 8.5±0.6 | 8.5±0.8 | 8.4±1.0 | 8.1±0.6 | 11.3±1.4 | 11.3±1.0 | 12.1±0.7 | 11.1±0.2 | 11.9±1.2 | 11.9±1.0 | 11.3±0.5 | 11.3±0.3 | 11.8±0.5 | 11.3±1.1 | 10.9±0.9 | 10.0±0.8 |
| Neut (10^3^/µL)  (1.4-7.3) | 4.3±1.6 | 4.4±1.4 | 5.4±2.0 | 4.4±1.1 | 4.4±2.9 | 3.4±1.3 | 4.4±2.5 | 3.7±1.1 | 3.6±0.9 | 5.3± 1.1 | 5.3±2.0 | 5.3± 1.0 | 3.2±1.3 | 4.1±0.2 | 6.6±1.5 | 4.7±2.1 |
| Lymp (10^3^/µL)  (2.3-13.0) | 2.9±0.9 | 2.9±1.0 | 2.8±1.2 | 2.5±1.0 | 3.4±0.5 | 3.3±1.1 | 4.0±1.2 | 3.9±1.8 | 2.9±0.6 | 3.1±1.0 | 3.2±0.6 | 3.6±0.2 | 3.2±1.0 | 3.0±0.8 | 3.4±1.3 | 3.7±1.5 |
| Mono (10^3^/µL)  (0.1-1.5) | 0.5±0.2 | 0.4±0.1 | 0.4±0.3 | 0.4±0.2 | 0.4±0.3 | 0.4±0.2 | 0.4±0.2 | 0.5±0.2 | 0.2±0.1 | 0.3±0.0 | 0.3±0.1 | 0.3±0.2 | 0.3±0.0 | 0.2±0.0 | 0.4±0.1 | 0.3±0.1 |
| Eos (10^3^/µL)  (0.0-0.8) | 0.2±0.1 | 0.3±0.2 | 0.3±0.3 | 0.2±0.2 | 0.0±0.0 | 0.0±0.0 | 0.0±0.0 | 0.0±0.0 | 0.0±0.0 | 0.0±0.0 | 0.0±0.0 | 0.0±0.0 | 0.0±0.0 | 0.0±0.0 | 0.0±0.0 | 0.1±0.0 |
| Baso (10^3^/µL)  (0.0-0.8) | 0.0±0.1 | 0.0±0.0 | 0.0±0.0 | 0.0±0.0 | 0.0±0.0 | 0.0±0.0 | 0.0±0.0 | 0.0±0.0 | 0.0±0.0 | 0.0±0.0 | 0.0±0.0 | 0.0±0.0 | 0.0±0.0 | 0.0±0.0 | 0.0±0.0 | 0.0±0.0 |
| n=12 for CoVLP ± adjuvant groups and n=8 for control group at pre-challenge (Day 7 post immunization). n=5 for CoVLP ± adjuvant groups at Day 6, n= 3 at Days 13 and 21. n=3 for control at Day 6, n=2 at Days 13 and 21. Data are presented as mean ± Standard Deviation | | | | | | | | | | | | | | | | |

#### **Supplementary Table 6:** **Peripheral hematology in rhesus macaques infected with SARS-CoV-2 after two immunizations with CoVLP vaccine, unadjuvanted or adjuvanted with AS03 or CpG 1018**

| **Parameter**  **(Normal values)** | **Pre-challenge** | | | | **Post-Challenge** | | | | | | | | | | | |
| --- | --- | --- | --- | --- | --- | --- | --- | --- | --- | --- | --- | --- | --- | --- | --- | --- |
|  |  |  |  |  | **Day 6** | | | | **Day 13** | | | | **Day 20 ^1^** | | | |
|  | **CoVLP** | **CoVLP + CpG 1018** | **CoVLP + AS03** | **Control** | **CoVLP** | **CoVLP + CpG 1018** | **CoVLP + AS03** | **Control** | **CoVLP** | **CoVLP + CpG 1018** | **CoVLP + AS03** | **Control** | **CoVLP** | **CoVLP + CpG 1018** | **CoVLP + AS03** | **Control** |
| WBC (10^3^/µL)  (6.6-15.5) | 7.0±1.8 | 7.0±2.5 | 7.2±1.9 | 8.3±1.8 | 9.8±3.8 | 8.3±2.2 | 9.6±2.3 | 8.4±3.0 | 11.3±4.2 | 9.3±3.0 | 7.9±2.5 | 9.1±2.9 | 9.1±0.7 | 8.4±1.3 | 10.4±2.6 | 9.8±0.3 |
| RBC (10^6^/µL)  (4.1-7.8) | 5.20±0.38 | 5.28±0.49 | 5.51±0.42 | 5.67±0.29 | 5.07±0.45 | 5.35±0.40 | 5.66±0.43 | 5.44±0.13 | 5.41±0.36 | 5.51±0.54 | 5.76±0.50 | 5.59±0.17 | 5.70±0.16 | 5.98±0.48 | 5.80±0.54 | 5.88±0.08 |
| HGB (gm/dL)  (10.1-15.9) | 11.9±0.4 | 12.2±0.8 | 12.3±1.0 | 13.5±0.7 | 11.8±0.7 | 12.5±0.9 | 12.7±0.8 | 13.3±0.7 | 12.4±0.4 | 12.9±0.8 | 13.1±0.8 | 13.5±0.1 | 13.0±0.5 | 14.2±0.6 | 13.3±0.9 | 14.3±0.2 |
| Hematocrit (%)  (34.8-55.2) | 37.4±1.5 | 38.0±2.5 | 38.5±2.8 | 41.6±1.5 | 35.8±2.0 | 37.7±2.5 | 38.9±2.5 | 39.0±1.4 | 36.8±1.7 | 38.8±2.8 | 39.5±2.7 | 39.6±0.1 | 38.8±1.2 | 42.2±2.7 | 39.7±3.0 | 41.9±1.0 |
| MCV (fL)  (63.7-86.9) | 72.2±3.2 | 72.1±2.9 | 69.9±2.1 | 73.6±1.9 | 70.7±3.4 | 70.7±1.7 | 68.8±1.1 | 71.6±1.8 | 68.2±1.7 | 70.6±2.1 | 68.6±1.5 | 70.9±1.9 | 68.0±1.7 | 70.6±1.7 | 68.6±1.5 | 71.4±2.6 |
| MCH (pg)  (19.1-27.7) | 23.0±0.9 | 23.2±1.0 | 22.4±0.9 | 23.8±0.9 | 23.3±1.1 | 23.5±1.0 | 22.5±0.9 | 24.4±0.8 | 22.9±1.0 | 23.5±0.9 | 22.7±0.7 | 24.1±0.6 | 22.8±0.9 | 23.7±1.1 | 23.1±1.0 | 24.3±0.6 |
| MCHC (gm/dL)  (28.9-35.4) | 31.8±0.4 | 32.2±0.3 | 32.0±0.3 | 32.4±0.6 | 32.9±0.6 | 33.2±0.7 | 32.8±1.0 | 34.0±0.6 | 33.6±0.6 | 33.3±0.2 | 33.2±0.4 | 34.0±0.1 | 33.5±0.5 | 33.6±0.8 | 33.6±0.8 | 34.0±0.3 |
| RDW (%)  (10.9-15.3) | 14.3±0.9 | 14.1±0.5 | 14.1±0.6 | 13.8±0.3 | 12.2±0.4 | 12.1±0.4 | 12.3±0.7 | 12.3±0.9 | 12.2±0.2 | 12.3±0.8 | 12.4±0.5 | 12.4±0.6 | 12.1±0.1 | 12.1±0.8 | 12.3±0.4 | 12.3±0.6 |
| Platelet (10^3^/µL)  (193.1-676.2) | 364±84 | 391±76 | 411±34 | 453±42 | 342±53 | 367±60 | 308±109 | 442±111 | 312±94 | 393±100 | 294±91 | 421±70 | 405±54 | 466±75 | 434±54 | 556±58 |
| MPV (fL)  (7.0-12.0) | 8.4±0.6 | 8.7±0.8 | 8.6±1.0 | 8.1±0.5 | 11.6±0.5 | 11.6±1.0 | 12.0±1.1 | 11.5±0.5 | 11.3±0.6 | 11.2±1.0 | 11.5±0.7 | 11.2±0.4 | 10.7±0.7 | 10.8±0.6 | 10.3±0.6 | 11.3±0.5 |
| Neut (10^3^/µL)  (1.4-7.3) | 3.6±1.0 | 3.8±2.4 | 4.2±1.6 | 5.1±1.8 | 4.8±2.3 | 4.8±1.6 | 5.5±2.1 | 4.9±2.4 | 5.9±1.5 | 6.8±2.3 | 4.1±1.8 | 6.5±2.0 | 5.4±1.6 | 5.7±1.7 | 6.8±3.0 | 6.5±0.5 |
| Lymp (10^3^/µL)  (2.3-13.0) | 2.7±0.9 | 2.5±0.6 | 2.3±0.7 | 2.6±0.7 | 4.1±1.6 | 2.9±0.8 | 3.6±1.1 | 2.8±0.6 | 4.8±2.5 | 2.2±0.8 | 3.5±1.9 | 2.4±1.0 | 3.2±0.7 | 2.3±0.5 | 3.3±1.1 | 3.0±0.7 |
| Mono (10^3^/µL)  (0.1-1.5) | 0.4±0.2 | 0.4±0.2 | 0.3±0.1 | 0.4±0.2 | 0.8±0.3 | 0.6±0.1 | 0.5±0.3 | 0.6±0.3 | 0.4±0.1 | 0.3±0.1 | 0.2±0.1 | 0.1±0.1 | 0.4±0.1 | 0.4±0.2 | 0.3±0.1 | 0.3±0.0 |
| Eos (10^3^/µL)  (0.0-0.8) | 0.3±0.1 | 0.2±0.1 | 0.4±0.2 | 0.2±0.1 | 0.0±0.0 | 0.0±0.0 | 0.0±0.0 | 0.0±0.0 | 0.1±0.1 | 0.0±0.0 | 0.1±0.1 | 0.0±0.0 | 0.1±0.1 | 0.0±0.0 | 0.0±0.0 | 0.1±0.0 |
| Baso (10^3^/µL)  (0.0-0.8) | 0.0±0.1 | 0.0±0.0 | 0.0±0.0 | 0.0±0.0 | 0.0±0.0 | 0.0±0.0 | 0.0±0.0 | 0.0±0.0 | 0.0±0.0 | 0.0±0.0 | 0.0±0.0 | 0.0±0.0 | 0.0±0.0 | 0.0±0.0 | 0.0±0.0 | 0.0±0.0 |
| n=6 for CoVLP ± adjuvant groups and n=4 for control group at pre-challenge (Day 7 after the second immunization) and Day 6 post-challenge. n=3 for CoVLP ± CpG 1018 groups, n=4 for CoVLP + AS03 group and n=2 for control group at Days 13 and 20 post infection.  Data are presented as mean ± Standard Deviation  ^1^Due to logistic reasons, animals were euthanized on Days 20, 21 or 23, results are presented as Day 20. | | | | | | | | | | | | | | | | |

#### **Supplementary Table 7: Peripheral blood chemistry in rhesus macaques infected with SARS-CoV-2 after one immunization with CoVLP vaccine, unadjuvanted or adjuvanted with AS03 or CpG 1018**

| **Parameters**  **(Normal values)** | **Pre-Challenge** | | | | **Post-Challenge** | | | | | | | | | | | |
| --- | --- | --- | --- | --- | --- | --- | --- | --- | --- | --- | --- | --- | --- | --- | --- | --- |
|  |  |  |  |  | **Day 6** | | | | **Day 13** | | | | **Day 20** | | | |
|  | **CoVLP** | **CoVLP + CpG 1018** | **CoVLP + AS03** | **Control** | **CoVLP** | **CoVLP + CpG 1018** | **CoVLP + AS03** | **Control** | **CoVLP** | **CoVLP + CpG 1018** | **CoVLP + AS03** | **Control** | **CoVLP** | **CoVLP + CpG 1018** | **CoVLP + AS03** | **Control** |
| CRP (mg/dL)  (1-3) | <0.2 | <0.2 | <0.2 | <0.2 | <0.5 | 0.5±0.1 | 0.5±0.1 | 0.5±0.1 | <0.5 | <0.5 | <0.5 | <0.5 | <0.5 | <0.5 | <0.5 | <0.5 |
| Glucose (mg/dL)  (48-99) | 64±8 | 65±5 | 68±8 | 70±10 | 60±5 | 55±7 | 65±17 | 59±7 | 61±5 | 63±8 | 74±11 | 66±8 | 54.0±9 | 53±6 | 57±5 | 50±4 |
| BUN (mg/dL)  (13-27) | 17±6 | 14±2 | 15±2 | 16±2 | 14.7±1.9 | 14.1±1.8 | 14.5±3.2 | 14.0±3.5 | 17.3±4.7 | 16.3±5.0 | 12.4±2.3 | 12.5±4.9 | 16.7±0.7 | 18.3±1.7 | 16.7±1.4 | 16.6±1.3 |
| Creatine (mg/dL)  (0.4-1.4) | 1.0±0.2 | 1.0±0.2 | 1.0±0.2 | 1.1±0.2 | 0.74±0.14 | 0.78±0.13 | 0.78±0.21 | 0.65±0.14 | 0.81±0.12 | 0.93±0.26 | 0.85±0.15 | 0.69±0.01 | 0.70±0.12 | 0.76±0.18 | 0.78±0.16 | 0.63±0.04 |
| BUN/Creatine ^1^  (11-60) | - | - | - | - | 21±7 | 18±3 | 19±5 | 22±6 | 21±3 | 17±1 | 15±0 | 18±7 | 24±3 | 24±4 | 22±5 | 27±1 |
| Sodium (mEq/L)  (144-160) | 147±1 | 147±2 | 148±2 | 145±7 | 148 ±2 | 150 ±1 | 150 ±2 | 150 ±2 | 148 ±2 | 149 ±3 | 150 ±1 | 148 ±3 | 148±2 | 149 ±1 | 149 ±1 | 152 ±1 |
| Potassium (mEq/L)  (3.3-6.4) | 3.9±0.4 | 3.9±0.2 | 3.8±0.3 | 4.1±0.3 | 3.8±0.2 | 3.9±0.2 | 3.9±0.2 | 4.0±0.1 | 3.7±0.1 | 3.7±0.2 | 3.7±0.2 | 3.7±0.1 | 3.8±0.4 | 4.0±0.3 | 4.0±0.1 | 4.2±0.1 |
| Chloride (mEq/L)  (106-117) | 108±2 | 108±3 | 108±2 | 105±5 | 105±2 | 107±3 | 106±2 | 107±3 | 105±1 | 107±3 | 107±1 | 106±1 | 105±1 | 108 ±1 | 107±1 | 109±2 |
| Phosphorus (mg/dL)  (3.4-7.5) | 5.2±1.1 | 5.3±1.2 | 5.0±1.1 | 5.6±1.3 | 5.5±1.5 | 5.3±1.1 | 5.8±1.2 | 5.9±1.4 | 5.6±1.5 | 6.1±1.1 | 6.2±1.2 | 6.4±1.3 | 5.7±1.4 | 5.4±0.3 | 6.2±0.5 | 6.6±0.2 |
| Calcium (mg/dL)  (9.4-12.2) | 9.5±0.4 | 9.6±0.3 | 9.5±0.5 | 9.7±0.7 | 9.4±0.4 | 9.5±0.4 | 9.4±0.3 | 9.5±0.7 | 9.5±0.4 | 9.6±0.3 | 9.6±0.4 | 9.0±0.0 | 9.7±0.2 | 9.6±0.2 | 9.5±0.5 | 9.7±0.2 |
| Tot. Protein (g/dL)  (5.9-7.8) | 7.4±0.4 | 7.2±0.3 | 7.2±0.3 | 7.3±0.5 | 6.4±0.4 | 6.5±0.5 | 6.6±0.3 | 6.6±0.2 | 7.0±0.3 | 6.9±0.3 | 7.0±0.1 | 6.8±0.4 | 6.9±0.1 | 6.4±0.3 | 6.6±0.2 | 6.8±0.2 |
| Albumin (g/dL)  (3.0-5.9) | 3.8±0.3 | 3.8±0.3 | 3.7±0.2 | 3.9±0.4 | 4.1±0.3 | 4.2±0.5 | 4.1±0.3 | 4.1±0.3 | 4.4±0.1 | 4.4±0.2 | 4.4±0.2 | 3.9±0.1 | 4.4±0.1 | 4.2±0.3 | 4.2±0.1 | 4.0±0.2 |
| Globulin (g/dL)  (1.9-3.9) | 3.5±0.3 | 3.4±0.2 | 3.5±0.2 | 3.4±0.3 | 2.3±0.3 | 2.3±0.2 | 2.5±0.4 | 2.5±0.4 | 2.6±0.2 | 2.4±0.1 | 2.6±0.2 | 2.9±0.6 | 2.5±0.0 | 2.2±0.1 | 2.4±0.2 | 2.8±0.4 |
| A/G Ratio  (0.5-3.5) | 1.1±0.1 | 1.1±0.1 | 1.1±0.1 | 1.1±0.1 | 1.8±0.3 | 1.8±0.3 | 1.7±0.3 | 1.7±0.3 | 1.7±0.1 | 1.8±0.1 | 1.7±0.2 | 1.4±0.3 | 1.8±0.1 | 1.9±0.2 | 1.8±0.2 | 1.4±0.3 |
| Tot. Bili (mg/dL)  (0.1-0.7) | 0.2±0.1 | 0.2±0.1 | 0.2±0.0 | 0.2±0.1 | 0.16±0.06 | 0.13±0.04 | 0.13±0.02 | 0.14±0.06 | 0.15±0.04 | 0.13±0.03 | 0.14±0.00 | 0.16±0.04 | 0.11±0.02 | 0.09±0.02 | 0.10±0.03 | 0.12±0.01 |
| LDH (U/L)  (129-644) | 274±98 | 272±76 | 306±144 | 234±65 | 701 ±178 | 676 ±245 | 591 ±153 | 701 ±121 | 486 ±121 | 451 ±103 | 475 ±153 | 495 ±63 | 512 ±153 | 504 ±67 | 449 ±78 | 557 ±391 |
| GGT (U/L)  (32-89) | 79±19 | 83±18 | 82±14 | 84±19 | 59±15 | 62±17 | 59±16 | 60±12 | 61±5 | 67±11 | 70±13 | 65±6 | 56±6 | 64±18 | 68±14 | 63±13 |
| Alk Phos (U/L)  (55-649) | 293±130 | 348±170 | 352±179 | 419±241 | 214 ±104 | 274 ±158 | 256 ±138 | 275 ±147 | 222 ±122 | 310 ±112 | 280 ±112 | 294 ±185 | 221 ±116 | 295 ±108 | 293 ±118 | 360 ±252 |
| ALT (U/L)  (20-126) | 41±14 | 44±16 | 52±22 | 46±15 | 19±5 | 20±4 | 22±7 | 22±4 | 15±5 | 19±4 | 22±5 | 21±0 | 16±5 | 18±2 | 21±5 | 20±2 |
| AST (U/L)  (25-120) | 23±7 | 21±5 | 25±11 | 26±10 | 40±22 | 34±8 | 31±6 | 34±6 | 26±2 | 28±2 | 27±6 | 32±7 | 32±6 | 36±8 | 30±9 | 34±4 |
| Cholesterol (mg/dL)^1^  (106-241) | - | - | - | - | 130±17 | 139±11 | 139±21 | 148±6 | 124±21 | 129±9 | 150±7 | 134±4 | 132±24 | 119±3 | 140±12 | 125±6 |
| Trig. (mg/dL)^1^  (29-96) | - | - | - | - | 42±7 | 47±10 | 46±18 | 51±5 | 51±8 | 51±5 | 55±17 | 38±8 | 60±7 | 39±13 | 55±7 | 54±47 |
| Pre-challenge: n=12 for CoVLP, CoVLP + CpG 1018 and CoVLP + AS03 groups, n=8 for control group (Day 7 post immunization). Post-infection study Day 6: n=6 for CoVLP, CoVLP + CpG 1018 and CoVLP + AS03 groups, n=4 for control group. Post-infection study Days 6 and 13: n=3 for CoVLP, CoVLP + CpG 1018 and CoVLP + AS03 groups, n=2 for control group. Data are presented as mean ± Standard Deviation ^1^ Values not available for pre-challenge testing. | | | | | | | | | | | | | | | | |

#### **Supplementary Table 8: Peripheral blood chemistry in rhesus macaques infected with SARS-CoV-2 after two immunizations with CoVLP vaccine, unadjuvanted or adjuvanted with AS03 or CpG 1018**

| **Parameters**  **(Normal values)** | **Pre-Challenge** | | | | **Post-Challenge** | | | | | | | | | | | |
| --- | --- | --- | --- | --- | --- | --- | --- | --- | --- | --- | --- | --- | --- | --- | --- | --- |
|  |  |  |  |  | **Day 6** | | | | **Day 13** | | | | **Day 20 ^1^** | | | |
|  | **CoVLP** | **CoVLP + CpG 1018** | **CoVLP + AS03** | **Control** | **CoVLP** | **CoVLP + CpG 1018** | **CoVLP + AS03** | **Control** | **CoVLP** | **CoVLP + CpG 1018** | **CoVLP + AS03** | **Control** | **CoVLP** | **CoVLP + CpG 1018** | **CoVLP + AS03** | **Control** |
| CRP (mg/dL)  (1-3) | <0.2 | <0.2 | <0.2 | <0.2 | 0.5±0.1 | <0.5 | <0.5 | <0.5 | <0.5 | <0.5 | <0.5 | <0.5 | <0.5 | <0.5 | <0.5 | <0.5 |
| Glucose (mg/dL)  (48-99) | 56±8 | 58±6 | 60±5 | 56±3 | 42±14 | 49±11 | 61±13 | 51±4 | 62±14 | 66±16 | 76±8 | 68±0 | 52±5 | 51±12 | 58±8 | 39±6 |
| BUN (mg/dL)  (13-27) | 14±4 | 14±1 | 15±3 | 14±2 | 16±4 | 17±2 | 16±4 | 18±3 | 13±2 | 14±2 | 13±3 | 15±0 | 15±3 | 18±1 | 16±3 | 17±2 |
| Creatine (mg/dL)  (0.4-1.4) | 1.0±0.1 | 1.0±0.2 | 1.0±0.2 | 1.1±0.2 | 0.7±0.1 | 0.8±0.2 | 0.9±0.1 | 0.9±0.1 | 0.7±0.1 | 0.9±0.2 | 0.9±0.2 | 0.8±0.1 | 0.6±0.1 | 0.8±0.2 | 0.8±0.2 | 0.7±0.0 |
| BUN/Creatine^2^  (11-60) | - | - | - | - | 24.3±6.7 | 21.0±2.8 | 18.8±4.9 | 21.0±4.7 | 19.0±4.6 | 16.3±1.5 | 14.5±2.6 | 17.0±1.4 | 27.0±7.9 | 24.0±7.9 | 21.5±8.7 | 23.0±4.2 |
| Sodium (mEq/L)  (144-160) | 149±1 | 148±2 | 149±1 | 149 ±2 | 149±2 | 149±1 | 151±2 | 151±1 | 148±2 | 148±1 | 150±2 | 150±0 | 150±0 | 151±1 | 151±1 | 151±0 |
| Potassium (mEq/L)  (3.3-6.4) | 3.7±0.3 | 3.8±0.1 | 4.1±0.4 | 4.2±0.2 | 3.9±0.3 | 3.8±0.1 | 3.8±0.3 | 4.1±0.2 | 3.6±0.2 | 3.8±0.1 | 3.7±0.3 | 3.8±0.1 | 4.2±0.2 | 4.4±0.2 | 4.1±0.3 | 4.3±0.0 |
| Chloride (mEq/L)  (106-117) | 109±1 | 108±2 | 109±1 | 108±2 | 107±2 | 107±2 | 107±2 | 108±3 | 107±2 | 106±2 | 107±1 | 108±2 | 108±1 | 108±1 | 108±3 | 108±2 |
| Phosphorus (mg/dL)  (3.4-7.5) | 5.4±1.0 | 5.1±1.4 | 5.5±0.8 | 4.9±1.2 | 4.7±0.9 | 5.3±0.8 | 5.4±0.9 | 5.2±1.3 | 5.4±0.9 | 5.5±0.6 | 6.2±0.6 | 6.4±0.1 | 7.1±1.7 | 7.1±1.1 | 6.2±0.9 | 7.3±0.2 |
| Calcium (mg/dL)  (9.4-12.2) | 9.4±0.4 | 9.4±0.2 | 9.7±0.4 | 10.1±0.1 | 9.5±0.3 | 9.6±0.4 | 9.8±0.1 | 9.9±0.2 | 9.6±0.3 | 9.7±0.3 | 9.7±0.2 | 9.9±0.2 | 9.7±0.6 | 9.9±0.7 | 9.9±0.4 | 10.5±0.3 |
| Tot. Protein (g/dL)  (5.9-7.8) | 7.3±0.6 | 7.0±0.1 | 7.2±0.3 | 7.6±0.3 | 6.5±0.4 | 6.6±0.3 | 6.7±0.3 | 6.9±0.3 | 6.5±0.6 | 6.8±0.2 | 6.8±0.2 | 7.0±0.3 | 6.5±0.7 | 7.0±0.3 | 6.7±0.4 | 7.2±0.2 |
| Albumin (g/dL)  (3.0-5.9) | 3.8±0.3 | 3.7±0.2 | 3.8±0.2 | 4.1±0.3 | 3.9±0.2 | 4.1±0.2 | 4.3±0.4 | 4.3±0.2 | 4.1±0.4 | 4.4±0.2 | 4.3±0.3 | 4.6±0.1 | 4.1±0.5 | 4.5±0.4 | 4.2±0.3 | 4.6±0.1 |
| Globulin (g/dL)  (1.9-3.9) | 3.5±0.5 | 3.3±0.2 | 3.5±0.2 | 3.5±0.2 | 2.6±0.3 | 2.5±0.2 | 2.5±0.2 | 2.6±0.2 | 2.4±0.2 | 2.4±0.0 | 2.5±0.2 | 2.5±0.2 | 2.4±0.2 | 2.6±0.3 | 2.5±0.3 | 2.6±0.1 |
| A/G Ratio  (0.5-3.5) | 1.1±0.1 | 1.1±0.1 | 1.1±0.1 | 1.2±0.1 | 1.5±0.2 | 1.7±0.2 | 1.8±0.2 | 1.7±0.1 | 1.7±0.1 | 1.8±0.1 | 1.7±0.3 | 1.9±0.1 | 1.7±0.1 | 1.8±0.3 | 1.7±0.3 | 1.8±0.0 |
| Tot. Bili (mg/dL)  (0.1-0.7) | 0.2±0.0 | 0.2±0.0 | 0.2±0.0 | 0.2±0.1 | 0.1±0.1 | 0.1±0.0 | 0.1±0.0 | 0.1±0.0 | 0.2±0.1 | 0.1±0.1 | 0.1±0.0 | 0.1±0.0 | 0.1±0.1 | 0.1±0.0 | 0.1±0.0 | 0.1±0.0 |
| LDH (U/L)  (129-644) | 303±56 | 328±63 | 323±81 | 241±18 | 916±276 | 804±96 | 814±283 | 834±417 | 384±186 | 468±265 | 478±207 | 525±380 | 519±123 | 608±278 | 871±256 | 925±315 |
| GGT (U/L)  (32-89) | 84±18 | 85±18 | 97±18 | 81±9 | 60±16 | 67±18 | 68±11 | 52±7 | 68±16 | 65±24 | 68±16 | 60±10 | 72±14 | 71±30 | 69±18 | 63±4 |
| Alk Phos (U/L)  (55-649) | 313±163 | 301±172 | 326±154 | 356±206 | 270±151 | 283±146 | 299±152 | 265±163 | 428±162 | 408±231 | 387±206 | 430±199 | 475±169 | 453±258 | 392±196 | 418±126 |
| ALT (U/L)  (20-126) | 46±12 | 60±19 | 61±36 | 48±9 | 21.8±2.4 | 28.8±13.7 | 22.8±3.9 | 21.3±1.7 | 23.0±1.0 | 19.0±3.5 | 21.5±3.9 | 18.5±0.7 | 26.7±3.8 | 21.3±3.5 | 21.8±3.3 | 18.0±5.7 |
| AST (U/L)  (25-120) | 29±9 | 30±5 | 29±9 | 25±4 | 39.0±6.2 | 39.8±6.8 | 38.7±8.4 | 39.0±5.6 | 25.3±4.7 | 24.0±1.7 | 26.8±5.9 | 26.0±4.2 | 42.0±17.3 | 36.7±7.8 | 45.5±5.4 | 37.5±3.5 |
| Cholesterol (mg/dL)^2^  (106-241) | - | - | - | - | 123.0±17 | 139.5±32 | 137.8±17 | 157.5±22 | 127.7±26 | 146.7±26 | 143.5±21 | 168.5±29 | 125.3±36 | 146.0±27 | 148.8±33 | 150.5±30 |
| Trig. (mg/dL)^2^  (29-96) | - | - | - | - | 48.8±8.9 | 40.8±9.3 | 45.0±11.1 | 48.8±8.7 | 56.0±11.5 | 53.0±11.5 | 60.8±25.9 | 49.5±6.4 | 66.3±17.4 | 56.0±14.1 | 48.5±18.3 | 57.0±32.5 |
| n=6 for CoVLP ± adjuvant groups and n=4 for control group at pre-challenge (Day 7 after the second immunization) and Day 6 post-challenge. n=3 for CoVLP ± CpG 1018 groups, n=4 for CoVLP + AS03 group and n=2 for control group at Days 13 and 20 post infection.  ^1^ Due to logistic reasons, animals were euthanized on days 20, 21 or 23, results are presented as Day 20.  ^2^ Values not available for pre-challenge testing. | | | | | | | | | | | | | | | | |
