# Supplementary material for: Safety, immunogenicity, and protection provided by unadjuvanted and adjuvanted formulations of a recombinant plant-derived virus-like particle vaccine candidate for COVID-19 in nonhuman primates
Source: Cell Mol Immunol. 2022 Jan 5;19(2):222–33. doi: 10.1038/s41423-021-00809-2 (PMC8727235; doi:10.1038/s41423-021-00809-2)
Supplement: Supplementary file 5 — Scoring charts [file 41423_2021_809_MOESM5_ESM.pdf]

Additional information file S1: Scoring charts

Physical Examination

Date: ____________________ **Cage-Side Assessment** Animal ID: ______________________

Parameter	Description	Score	
Responsiveness	Normal - bright, alert, responsive	0	
	Mildly affected - slightly depressed, acts disinterested with personnel in room, lies down in cage but gets up when approached	1	
	Moderately affected/obtunded - non-responsive, very disinterested in personnel, hunched or lying down, will get up when stimulated	2	
	Severely affected/comatose - lying down completely unresponsive to stimuli	3	
Discharges	Normal	0	
	Mild nasal/ocular	1	
	Severe nasal/ocular	3	
Skin	Normal	0	
	Mild dermatitis	1	
	Severe dermatitis	3	
Respiratory	Normal - no apparent changes in breathing, (est 16-54 BPM), no cough	0	
	Mild - slightly increased effort breathing, (est 55-66 BPM), and/or mild cough	1	
	Moderate - obvious difficulty breathing, (est 67-80 BPM), and/or moderate cough	2	
	Severe - open mouth breathing, abdominal breathing, (est >80 BPM), cyanosis, and/or severe cough	3	
Food consumption	<25% of food remaining	0	
	≤25%-50% of food remaining	1	
	>50% of food remaining	2	
	Normal	0	
	Soft	1	

Fecal consistency	Fluid	2	
		**Total**	
**Notes** *(any observed sneezing, vomit, conjunctival erythema, or other abnormalities*)			

Date: ________________ **Physical Examination Under Anesthesia** Animal ID: _________________

Parameter	Description	Score	
Rectal temperature *(taken immediately after anesthesia induction)*	Normal (100.0-102.4F)	0	
	Mild hypothermia (98.0-99.9F)	1	
	Moderate hypothermia (96.0-97.9F)	2	
	Severe hypothermia (≤96.0)	3	
	Mild hyperthermia (102.5-103.4F)	1	
	Moderate hyperthermia (103.5-104.4F)	2	
	Severe hyperthermia (>104.4F)	3	
Respiratory rate	Normal (16-54 BPM)	0	
	Mild tachypnea (55-66 BPM)	1	
	Moderate tachypnea (67-80 BPM)	2	
	Severe tachypnea (>80 BPM)	3	
Respiratory character	Normal	0	
	Mild dyspnea	1	
	Severe dyspnea	3	
Auscultation	Normal	0	
	Mild (occasional crackles/rales or wheezes)	1	
	Severe (continuous crackles/rales or wheezes)	3	
SpO_2	Normal (96-100%)	0	
	Mildly decreased (92-95)	1	
	Moderately decreased (80-91)	2	
	Severely decreased (<80%)	3	
	Normal (0-4.9% loss)	0	

Body weight	Mild (5-10.9% loss)	1	
	Moderate (11-24.9% loss)	2	
	Severe (≥25% loss)	3	
Hydration	Normal (normal skin turgor, moist mucous membranes)	0	
	Mild dehydration (5-10%)	1	
	Severe dehydration (>10%)	3	
**Notes**		**Total**	
